# Leveraging gene co-expression patterns to infer trait-relevant tissues in genome-wide association studies

**DOI:** 10.1371/journal.pgen.1008734

**Published:** 2020-04-20

**Authors:** Lulu Shang, Jennifer A. Smith, Xiang Zhou

**Affiliations:** 1 Department of Biostatistics, University of Michigan, Ann Arbor, MI, United States of America; 2 Department of Epidemiology, University of Michigan, Ann Arbor, MI, United States of America; 3 Survey Research Center, Institute for Social Research, University of Michigan, Ann Arbor, MI, United States of America; 4 Center for Statistical Genetics, University of Michigan, Ann Arbor, MI, United States of America; Case Western Reserve University, UNITED STATES

## Abstract

Genome-wide association studies (GWASs) have identified many SNPs associated with various common diseases. Understanding the biological functions of these identified SNP associations requires identifying disease/trait relevant tissues or cell types. Here, we develop a network method, CoCoNet, to facilitate the identification of trait-relevant tissues or cell types. Different from existing approaches, CoCoNet incorporates tissue-specific gene co-expression networks constructed from either bulk or single cell RNA sequencing (RNAseq) studies with GWAS data for trait-tissue inference. In particular, CoCoNet relies on a covariance regression network model to express gene-level effect measurements for the given GWAS trait as a function of the tissue-specific co-expression adjacency matrix. With a composite likelihood-based inference algorithm, CoCoNet is scalable to tens of thousands of genes. We validate the performance of CoCoNet through extensive simulations. We apply CoCoNet for an in-depth analysis of four neurological disorders and four autoimmune diseases, where we integrate the corresponding GWASs with bulk RNAseq data from 38 tissues and single cell RNAseq data from 10 cell types. In the real data applications, we show how CoCoNet can help identify specific glial cell types relevant for neurological disorders and identify disease-targeted colon tissues as relevant for autoimmune diseases.

## Author summary

Identifying trait-relevant tissues or cell types is important for understanding disease etiology. Several computational methods have been recently developed to integrate omics studies with genome-wide association studies (GWASs) in order to infer trait-relevant tissues or cell types. For example, both LDSC-SEG and RolyPoly rely on genes that are specifically expressed in a given tissue or cell type for inferring trait-tissue relevance. However, these previous methods have thus far ignored an important biological feature of gene expression data; that is, genes are interconnected with each other and are co-regulated together. Such gene co-expression pattern occurs in a tissue specific or cell type specific fashion and may contain invaluable information for inferring trait-tissue relevance. Here, we develop a network model to take advantage of the tissue-specific or cell type specific gene co-expression patterns inferred from bulk RNA sequencing or single cell RNA sequencing studies into GWASs. We illustrate the benefits of our method in identifying trait-relevant tissues or cell types through simulations and applications to real data sets.

## Introduction

Genome-wide association studies (GWASs) have identified many SNPs associated with common diseases and disease related complex traits. However, over 90% of these identified associations reside in noncoding regions and have unknown biological function [1]. Characterizing the biological functions of these identified associations requires the identification of trait-relevant tissues, as the SNP effects on most traits likely act in a tissue-specific fashion [2, 3]. For example, it is well recognized that brain-specific SNP effects underlie many brain related diseases such as psychiatric disorders [4–7]. For most complex traits, however, their trait-relevant tissues are often obscure. As a result, identifying trait-relevant tissues from GWAS becomes an important first step towards understanding disease etiology and the genetic basis of phenotypic variation [8–15].

Recent development of RNA sequencing (RNAseq) technology, including both bulk RNAseq and scRNAseq, have provided complementary information for the inference of disease relevant tissues. These RNAseq studies produce accurate gene expression measurements both at a genome-wide scale and in a tissue specific or cell type specific fashion. For example, the Gene-Tissue Expression (GTEx) project performs bulk RNAseq to collect gene expression measurements from hundreds of individuals across ~50 tissues [16]. More recently, various scRNAseq studies are being performed to collect cell type specific gene expression measurements on tens of thousands of cells from various tissues and organs [17]. Such tissue specific and cell type specific expression measurements collected from bulk RNAseq and scRNAseq provide valuable information for inferring disease relevant tissue types. Indeed, methods have been developed to identify genes that are specifically expressed in a particular tissue or cell type to construct tissue specific or cell type specific annotations at the gene level, which are further integrated into GWASs to infer disease-relevant tissues or cell types[18, 19]. However, these previous methods have ignored an important biological feature of gene expression data; that is, genes are interconnected with each other and are co-regulated together. Such gene co-expression patterns occur in a tissue specific or cell type specific fashion [8]. Certain gene co-expression sub-networks have been shown to contain valuable information for predicting gene-level association effect measurements on diseases in GWASs [8, 20–22]. In addition, genes with high network connectivity are enriched for heritability of common diseases and disease related traits [23]. Indeed, one key hypothesis in the recent omnigenic model states that tissue-specific gene networks underlie the etiology of various common diseases [24]. Therefore, it is important to develop statistical methods that can take advantage of tissue-specific topological connections contained in tissue specific gene co-expression networks to facilitate the inference of disease tissue relevance.

Here, we make such a first attempt to integrate GWAS data with gene co-expression patterns obtained from gene expression studies, through developing a statistical method for the inference of trait-relevant tissues. To do so, for a given trait, we treat the gene-level association statistics obtained from GWAS as the outcome variable and treat the tissue-specific adjacency matrices inferred from gene expression studies as input matrices. We examine one tissue at a time and model the gene-level association statistics as a function of the tissue-specific adjacency matrix. Afterwards, we identify trait-relevant tissues through likelihood-based inference. To accompany our model, we also develop a composite likelihood-based algorithm, which is computationally efficient and ensures result robustness in the presence of substantial noise in the estimated tissue-specific gene adjacency matrices. We refer to our method as *Co*mposite likelihood-based *Co*variance regression *Net*work model, or CoCoNet. We demonstrate the effectiveness of our method through simulations. We apply our method for an in-depth analysis of four autoimmune diseases and four neurological disorders, through integrating the corresponding GWASs with bulk RNAseq data from 38 tissues and single cell RNAseq data from 10 cell types.

## Materials and methods

### Covariance regression network model: The simplified version

In this section, we provide a simplified version of the covariance regression network model, which is used in most analyses in the present study. The general version of the covariance regression network model is described in the next section.

Here, we aim to leverage tissue-specific gene co-expression networks to infer trait relevant tissues through integrating GWAS and gene expression studies. To do so, we perform gene-centric analysis and focus on a common set of *m* genes that are measured in both GWAS and gene expression studies. For these genes, we obtain an *m*-vector of summary statistics in terms of gene-level effect measurements from GWAS for the trait of interest and denote the vector as ***y*** = (*y*_1_,⋯,*y*_*m*_)^*T*^. We also obtain tissue-specific gene expression measurements from gene expression studies on multiple tissues. For each tissue in turn, we construct an *m* by *m* gene-gene adjacency matrix to represent the co-expression network. Such adjacency matrix is constructed based on gene expression measurements, paired with prior gene-gene interaction information obtained from external data sources (more details in the following subsections). We denote the constructed tissue-specific symmetric adjacency matrix as ***A*** = (*a*_*ij*_), where its *ij*’th element *a*_*ij*_ is one if gene *i* is connected to gene *j* in the network and is zero otherwise. We set *a*_*ii*_ to be zero for any 1≤*i*≤*m* to ensure the absence of self-loops [25].

We reason that, in the trait-relevant tissue, if two genes share similar functionality, then these two genes will likely have similar effects on the trait of interest. In contrast, in the trait irrelevant tissue, two genes sharing similar functionality would not be strongly predictive of their effect measurement similarity on the trait of interest. Therefore, the prediction ability of the adjacency matrix ***A*** on the gene-level effect measurement *y*_*i*_ would be an effective indication on whether the examined tissue is relevant to trait or not. To capture such intuition, we use the Covariance Regression Network Model [26] to model the relationship between ***A*** and ***y***. Specifically, we consider

$$y∼MVN(1_{m}\mu ,\Sigma (A))$$ (1)

where **1**_*m*_ is an m-vector of 1s; *μ* is the intercept; MVN denotes multivariate normal distribution; and Σ(***A***) is the covariance of ***y*** that is a function of the adjacency matrix ***A***. In the simplified version, we consider $\Sigma (A)=\sigma_{1}^{2}A+\sigma_{0}^{2}I$. We also define $\rho =\sigma_{1}^{2}/(\sigma_{0}^{2}+\sigma_{1}^{2})$, which represents the relative signal strength of gene co-expression pattern on gene-level effect measurements ***y***. An extension of the model is shown in the next section.

The above model can be fitted through a standard maximum likelihood inference procedure. However, parameter estimation through the standard maximum likelihood inference procedure is computationally inefficient as it scales cubically with the number of genes *m*. To enable scalable computation, we consider the composite likelihood approach for inference [27]. Specifically, instead of working on the joint likelihood specified in Eq (1), we consider pairs of genes one at a time. For each pair of genes *i* and *j*, we consider the pair-wise likelihood $P(y_{i},y_{j}|\mu ,\sigma_{0}^{2},\sigma_{1}^{2})$ as

$$\left(\begin{array}{c} y_{i}\\ y_{j} \end{array}\right)∼BN(\left(\begin{array}{c} \mu \\ \mu \end{array}\right),\sigma_{0}^{2}\left(\begin{array}{cc} 1 & 0\\ 0 & 1 \end{array}\right)+\sigma_{1}^{2}\left(\begin{array}{cc} a_{ii} & a_{ij}\\ a_{ij} & a_{jj} \end{array}\right))$$ (2)

where *BN* denotes a bivariate normal distribution. With the model specified in Eq (2), we can obtain the corresponding log composite likelihood as:

$$l(\theta )=\sum_{i=1}^{m}\Sigma_{j>i}^{m}logP(y_{i},y_{j}|\mu ,\sigma_{0}^{2},\sigma_{1}^{2})$$ (3)

We use the Nelder-Mead method implemented in the optim function in R to obtain the composite likelihood estimates $\left(\hat{\mu },{\hat{\sigma }}_{0}^{2},{\hat{\sigma }}_{1}^{2}\right)$ that maximizes the above composite likelihood. Our inference algorithm scales only quadratically with the number of genes, and, with a small *K*, can analyze each trait-tissue pair with 10,000 genes in a few minutes.

Besides scalable computation, we note that the composite likelihood-based inference algorithm also ensures result robustness with respect to model mis-specifications. Specifically, instead of making the strong assumption that the *m*-vector of ***y*** jointly follows a multivariate Gaussian distribution, our composite likelihood only needs to make a much weaker assumption that each pair (*y*_*i*_,*y*_*j*_) follows a bivariate normal distribution. As a result, the composite likelihood-based algorithm can be robust to various model misspecifications, relieving a potential concern in real data applications, where the tissue specific adjacency matrices may be estimated with a substantial estimation noise.

We refer to the above model as the simplified version of the *Co*mposite likelihood-based *Co*variance regression *Net*work model, or CoCoNet. With the composite likelihood inference algorithm, we examine one tissue at a time and obtain the maximum composite likelihood. Afterwards, we rank the tissues based on the maximum composite likelihood and select the tissue with the highest likelihood as the most trait-relevant tissue. Because of the composite likelihood approach, CoCoNet is computationally efficient and can analyze each trait-tissue pair in real data in minutes (S1 Table). The CoCoNet method is implemented as an R package, which, together with all processed data and scripts to reproduce the results in the paper, are freely available at www.xzlab.org/software.html.

### Covariance regression network model: The extension

In the previous section, we have only focused on the simple case of $\Sigma (A)=\sigma_{1}^{2}A+\sigma_{0}^{2}I$. Here, we consider its natural extension. To do so, we denote $A^{k}=(a_{ij}^{\left(k\right)})$ as the *k-*th power of ***A***, for any integer *k*. It can be easily shown that *a*_*ij*_^(*k*)^ is the number of *k*-paths linking from gene *i* to gene *j* in the co-expression network, where *k*-paths are any paths of length *k*. For example, when *k* = 2, $a_{ij}^{\left(2\right)}=\sum_{h=1}^{m}a_{ih}a_{hj}$, where *a*_*ih*_*a*_*hj*_ is one only when there is a link connecting the three genes *i*−*h*−*j* and is zero otherwise. We also set $a_{ii}^{\left(k\right)}$ to be zero for *k*≥1. When *k* = 0, we define ***A***^0^ = ***I***, which is an *m*-dimensional identity matrix.

Following [26], we consider the use of polynomial matrix functions for the construction of Σ(***A***) by setting $\Sigma (A)=\sum_{k=0}^{K}\sigma_{k}^{2}A^{k}$, where *K* is the maximum number of paths linking between two genes considered in the model and is treated as a pre-fixed parameter. Intuitively, $\sigma_{1}^{2}A$ captures the gene-level effect measurement correlation due to direct connections among genes, while $\sigma_{k}^{2}A^{k}(k>1)$ captures the gene-level effect measurement correlation due to indirect connections among genes (i.e. gene-gene connection through other genes).

With the extension of Σ(***A***), the Eqs (2) and (3) becomes:

$$\left(\begin{array}{c} y_{i}\\ y_{j} \end{array}\right)∼BN\left(\left(\begin{array}{c} \mu \\ \mu \end{array}\right),\sum_{k=0}^{K}\sigma_{k}^{2}(\begin{array}{cc} a_{ii}^{\left(k\right)} & a_{ij}^{\left(k\right)}\\ a_{ij}^{\left(k\right)} & a_{jj}^{\left(k\right)} \end{array})\right),$$ (4)

and

$$l(\theta )=\sum_{i=1}^{m}\Sigma_{j>i}^{m}\log P(y_{i},y_{j}|\mu ,\sigma_{0}^{2},\cdots ,\sigma_{k}^{2}).$$ (5)

In the above model, the number of covariance matrices used, *K*, is treated as a fixed parameter. In the real data applications, we explored a few different choices of *K* in the range between one and four. We found that models with small *K* values are often preferred based on Bayesian Information Criterion (BIC): in all of the trait-tissue pairs, a model with *K* = 1 has the smallest BIC (S1 Fig). The lower BIC in models with small *K* is presumably because the direct connections described in ***A*** contain most information for predicting the correlation among gene-level effect measurements. Therefore, due to convenience and practical effectiveness, we focus on modeling with *K* = 1 (i.e. the simplified version of CoCoNet) in all our simulations and real data applications.

We note, however, that our CoCoNet model and software implementation are general: it applies to any pre-specified *K* and can also perform model selection to determine the optimal choice of *K*. Indeed, the high order (K>1) terms have nature biological interpretations of representing the high order connection among genes. Subsequently, even though these high order terms do not appear to have added benefits for the particular data sets we examined in the manuscript and/or for the particular application we considered, these terms might be suitable for many other genetics applications that we did not explore. For example, CoCoNet or some simple extension of it may be directly used in gene expression studies for predicting disease status using gene network information (e.g. applications like [28]) or using image information (e.g. applications like [29]); or for integrating chromatin contact information such as HiC data in different tissues or cell lines for understanding disease etiology [30]. In all these cases, it is possible that higher order terms may help improve prediction or inference accuracy.

### Simulations

We performed simulations to examine the effectiveness of our method. To do so, we first randomly selected 10 tissues and 1,000 genes from the genotype-tissue expression (GTEx) study. We then used these gene expression data to construct tissue-specific gene adjacency matrices, with which we further simulated gene level effects sizes as outcomes. Specifically, for each tissue in turn, we first categorized the selected 1,000 genes into non-overlapping gene clusters using the *k*-means clustering algorithm. The number of clusters for each tissue was determined based on BIC and ranged from 10 to 15 across tissues. Based on the gene clusters, we constructed the tissue-specific gene adjacency matrix as a block diagonal matrix: two genes are adjacent to each other if both belong to the same inferred cluster and are not adjacent to each other otherwise. Note that we constructed the gene adjacency matrices in a simpler way in the simulations than in the real data (details of how gene adjacency matrices are constructed in the real data are provided in the following section), to ensure that the covariance matrices are positive definite in the simulations so that we can easily simulate outcome variables from multivariate normal distributions. Next, we denoted one of the 10 tissues as the trait-relevant tissue and used its tissue-specific adjacency matrix to simulate our outcome variables. Afterwards, we fit data using the 10 tissues one at a time to identify the trait-relevant tissue. We examined three main simulation scenarios, each consisting of multiple parameter settings. We performed 100 simulation replicates for each parameter setting. We computed power as the percent of replicates where the true trait relevant tissue is correctly identified.

### Scenario I

The first simulation scenario is based on our model and assumes that we can directly observe the gene adjacency matrices for all tissues. Here, in each simulation replicate, we randomly designated one tissue out of the 10 tissues to be the trait-relevant tissue. We simulated the outcome variables through a multivariate normal distribution with mean zero and covariance matrix as $A\sigma_{1}^{2}+I\sigma_{0}^{2}$, where ***A*** is the adjacency matrix from the designated trait-relevant tissue. In the simulations, we set $\sigma_{1}^{2}+\sigma_{0}^{2}=1$ and varied $\rho =\sigma_{1}^{2}/(\sigma_{1}^{2}+\sigma_{0}^{2})$ in the range of 0 to 0.09 to examine the influence of signal strength on power. Note that *ρ* = 0.02 is close to the median estimate in the real data applications. In each replicate, we applied our model to examine one tissue at a time, treated the tissue-specific adjacency matrices as observed, and selected among the 10 tissues the one with the highest log likelihood (equivalently the lowest BIC) as the trait-relevant tissue.

### Scenario II

The simulation scenario II is similar to scenario I, except that we were unable to observe the true adjacency matrices. Instead, we were able to observe only a noisy version of the adjacency matrices. Specifically, we simulated the data using the true tissue-specific adjacency matrix as done in scenario I. However, when we fitted data, we were only provided with the observed tissue-specific adjacency matrices that were a noisy version of the truth. To generate these observed adjacency matrices, for each tissue in turn, we randomly converted a proportion *p* of the adjacent gene pairs in the true adjacency matrix to be nonadjacent, and randomly converted a proportion *q* of nonadjacent gene pairs in the true adjacency matrix to be adjacent. For each value of *p*, we chose the value of *q* so that the total number of adjacent gene pairs was the same between the true adjacency matrix and the observed adjacency matrix. Here, we fixed *ρ* = 0.02, a value close to the median estimate in the real data application; and we varied *p* from 0 to 0.9 to capture an increasingly large measure of noise.

### Scenario III

The simulation scenario III is similar to scenario II, except that the number of adjacent gene pairs in the observed adjacency matrices is much larger than the number of adjacent gene pairs in the true adjacency matrices, potentially reflecting an over-estimation of the number of adjacent gene pairs that may be observed in some real data applications. The observed adjacency matrices in this scenario was created by adding new adjacent gene pairs to the true adjacency matrices. Specifically, for each tissue in turn, we randomly converted a proportion *q* of nonadjacent gene pairs in the true adjacency matrix to be adjacent in the observed adjacency matrix. The adjacent genes in the true adjacency matrix serve as core genes that truly influence the gene-level association evidence with the complex trait. We again simulated the data using the true adjacency matrix and fitted data using the observed adjacency matrices. Here, we fixed *ρ* = 0.02, a value close to the median estimate in the real application; and we varied *q* from 0 to 0.9 to capture a range of core gene set proportions.

### Real data sets

#### Gene-level effect measurements from GWASs

We examined a total of eight disease traits from existing large-scale GWASs. These traits include four neurological disorders and four autoimmune disorders, with GWAS sample sizes ranging from 13,239 to 70,100. The examined neurological disorders include schizophrenia (SCZ) [31], bipolar disorder/schizophrenia (BIPSCZ) [32], bipolar disorder (BIP) [32], and Alzheimer’s disease [33], where BIPSCZ includes combined individuals from SCZ and BIP. The examined autoimmune disorders include primary biliary cirrhosis (PBC) [34], ulcerative colitis (UC) [35], inflammatory bowel disease (IBD) [35], and Crohn’s disease (CD) [35]. We selected these traits because the trait-relevant tissues for both neurological disorders and autoimmune disorders are relatively clear and because these traits are measured on GWASs with at least 12,000 samples, ensuring sufficient power [36]. The information for the summary statistics of eight GWAS traits can be found in S2 Table.

For each of these traits, we first obtained SNP-level summary statistics in the form of marginal z-scores. Following [8], we removed SNPs within the major histocompatibility complex (MHC) region (Chr6: 25Mb- 34Mb). We intersected the remaining SNPs from all eight studies to retain a common set of 622,026 SNPs for analysis. Besides SNP-level marginal z-scores, we also obtained individual-level genotypes of 503 European individuals from the 1000 Genomes Project to serve as a reference panel for linkage disequilibrium (LD) computation [37]. In addition, we obtained location information for 51,014 genes from the GENCODE project [38], and extracted cis-SNPs that reside within 1Mb before the transcription start site (TSS) and within 1Mb after the transcription end site (TES). We focused on 49,015 genes that have at least 10 SNPs, with an average of 438.8 SNPs in each gene (median = 448; min = 10; max = 2,425). Afterwards, with SNP-level statistics and LD information from the reference panel, we obtained gene-level heritability estimates using MQS [39]. We scaled the gene-level heritability estimates by the number of SNPs in each gene. Afterward, we further standardized the scaled values across genes to have a mean of zero and standard deviation of one. These final values are served as the gene level effect measurements for the GWAS trait.

#### Tissue specific gene co-expression networks from GTEx

We obtained tissue-specific gene co-expression networks inferred based on bulk RNAseq data collected on 38 tissues in the GTEx project [40] from https://zenodo.org/record/838734#.XALkry3MxTZ. Details on how these tissue-specific gene co-expression networks were constructed are described in [41]. Briefly, these networks were constructed using the software PANDA (Passing Attributes between Networks for Data Assimilation) [42], relying on information extracted from both gene expression measurements and an existing database that contains both known transcription factor (TF)-target gene interactions and known protein-protein interactions. We intersected the set of genes in the aforementioned database with the set of genes from GWASs to obtain an overlapping set of 25,991 genes for analysis. In the networks constructed through PANDA, the genes are represented as nodes while the connected gene pairs are represented as edges. Each gene and each edge have a specificity score calculated in each tissue. Following [41], we focused our analysis on the edges that are identified to be specific in at least one tissue, and genes that are specific (i.e. with a non-zero value) in at least two tissues (details in [41]). In addition, we included genes that are TF factors based on the database and have at least one connection to genes that we already retained. We focused our analysis on a final set of 5,359 genes. The edge value between a pair of genes represents evidence on whether the given gene pair is connected in the network or not: a strongly positive edge value indicates greater evidence on the existence of gene-gene connection. The edge values across all gene pairs have an approximate mean value of zero and a standard deviation of one. We then used the forceSymmetric function in R to ensure matrix symmetry. For edge values, we performed a hard thresholding procedure to convert the continuous edge values to binary values. In particular, edges that had a positive edge value and that were specific to at least one tissue type were converted to one; otherwise, they were converted to zero. This way, tissue-specific gene co-expression networks were converted to tissue-specific adjacency matrices used in our model.

We examined the sensitivity of the trait-tissue inference results with respect to how the adjacency matrices are constructed. Specifically, we varied the hard cut-off thresholds in converting the gene co-expression network inferred from PANDA to the adjacency matrices to be either -1, -0.5, 0, 0.5, or 1. We explored approaches of using only gene pairs (i.e. edges) that are not specific to any tissue or using only genes (i.e. nodes) that are non-specific to any tissues to build the gene co-expression network with PANDA. We also used the WGCNA software [43] to infer the tissue specific networks in place of PANDA. WGCNA does not require the additions of TF binding information or protein-protein interaction information. In fitting WGCNA, we set the soft thresholding power to be 6, converted edges with values greater than 0.005 to be 1, and converted edges with values smaller than 0.005 to be 0.

In addition to the main analysis, we conducted a side analysis by including a gene distance matrix as a covariance component in CoCoNet to control for potential gene distance confounding. To do so, we measured the distances between the transcript starting site (TSS) of any pairs of genes. We then scaled these distance measurements by the maximum value to be within the range of 0 and 1. Finally, we set distance to be one for pairs of genes that reside on different chromosomes.

#### Cell type specific gene co-expression networks from GTEx

We obtained single cell nucleus sequencing data with droplet technology (DroNc-seq) generated on archived frozen adult human post-mortem tissues from the GTEx project [44]. The GTEx DroNc-seq data contains gene expression measurements on 32,111 genes and 14,963 single cells from adult frozen human hippocampus (Hip, 4 samples) and prefrontal cortex (PFC, 3 samples) from five donors. We retained 16,930 genes that overlapped with genes from the GWASs. For each gene in turn, we transformed the data in the unit of reads per kilobase million (RPKM), and performed log10 transformation on the RPKM values after adding a constant of one. Following [45], we normalized the expression values across genes in each cell to a standard normal distribution, and further normalized the expression values of each gene across cells to a standard normal distribution. All cells in the data were already clustered into 10 cell types in the original paper using the *k*-nearest neighbor (*k*-NN) method [44]. These 10 cell types are exPFC, which consists of excitatory glutamatergic neurons in the prefrontal cortex; GABA, which consists of GABAergic interneurons; exCA, which consists of excitatory pyramidal neurons in the hippocampal Cornu Ammonis (CA) region; exDG, which consists of excitatory granule neurons from the hippocampal dentate gyrus region; ASC, which consists of astrocytes; MG, which consists of microglia cells; ODC, which consists of oligodendrocytes; OPC, which consists of oligodendrocyte precursor cells; NSC, which consists of neuronal stem cells; and END, which consists of endothelial cells. Following [41], we used the software PANDA to infer cell type specific gene co-expression networks for these 10 different cell types in each of the two donors separately. Because the gene co-expression networks for the same cell type in the two donors are similar to each other, we merged the inferred gene regulatory networks for the same cell type from the two donors together by taking the unions of the two corresponding gene co-expression networks. In the constructed networks, we calculated gene specificity scores (details in [41]) and retained half of the genes with a total specificity score across tissues above the median value. In addition, we retained genes that are either TF factors or have at least one connection to genes we already retained. This way, we retained a total of 8,269 genes for final analysis. We created cell type specific gene co-expression adjacency matrices for these genes based on the inferred co-expression networks following the same procedure described in the previous section.

#### Relevance between traits and tissues/cell types from PubMed search

Following [8], we partially validated the identified trait-relevant tissue/cell types for the GWAS diseases by searching PubMed. We reasoned that, if the tissue or cell type is relevant to the disease of interest, then there would be previous publications studying the disease in the particular tissue or cell type. Therefore, by counting the number of previous publications using the key word pairs of trait and tissue or the key word pairs of trait and cell type, we would have a reasonable quantitative estimate on the relevance between the trait and the corresponding tissue/cell type. To do so, we counted the number of references on the disease and tissue pairs for the 8 GWAS diseases and 38 tissues. In addition, we also counted the number of references on the disease and cell type pairs for the 4 neurological disorders and 10 brain cell types. We used an R package RISmed (https://cran.r-project.org/web/packages/RISmed/index.html) to efficiently count the number of publications in PubMed that contain the names of both the trait and the tissue/cell type either in the abstract or in the title [46]. The keywords of traits and tissues/cell types that we used in searching are listed in the supplementary S3–S5 Tables. For example, for the trait-tissue pair of schizophrenia and cerebellum, we conducted the search by using “Schizophrenia[Title/Abstract] AND cerebellum[Title/Abstract]”, which yielded 946 hits. After obtaining the number of papers on each trait-tissue/cell type pair, for each trait at a time, we further normalized the count data across tissues or across tissues/cell types by calculating the percentage of publications on each tissue or on each tissue/cell type.

#### Inferring trait relevant tissues/cell types with RolyPoly and LDSC-SEG

We compared our results with two existing methods that use gene-level annotations to infer trait relevant tissue/cell types: RolyPoly and LD score regression (LDSC-SEG). RolyPoly requires input that include GWAS summary statistics, gene expression profiles, an expression data annotation file, and linkage disequilibrium (LD) information. For GWAS summary statistics and gene expression profiles, we used the same input data for RolyPoly as used for our analysis. To make the gene annotation file, we defined a block as a 10kb window centered around each gene’s transcription start site (TSS) as recommended by RolyPoly. For LD information, we used the LD information provided by RolyPoly website, which were based on the 1000 genomes project phase 3 data and which set SNP pair covariance to be zero if below 0.2. For LDSC-SEG, following [19], we calculated *t*-statistics for each gene being differentially expressed in a given tissue or cell type versus all other tissue/cell types that are not in the same tissue category. For example, for cerebellum, we compared expression in cerebellum samples versus expression in all other samples but excluding the other brain regions. We then selected the top 2,000 tissue/cell type specific genes ranked by *t*-statistics. For these 2,000 genes, following [19], we annotated SNPs within 100-kb of their transcribed regions to have an annotation value of one and annotated the remaining SNPs to have an annotation value of zero. We then ran LDSC-SEG to estimate the heritability enrichment using the SNP annotation for each of the eight GWAS traits separately. Both RolyPoly and LDSC-SEG output *p* value for each examined trait-tissue pair, with which we ranked tissues for each trait.

## Results

### Method and simulations

**Fig 1:**
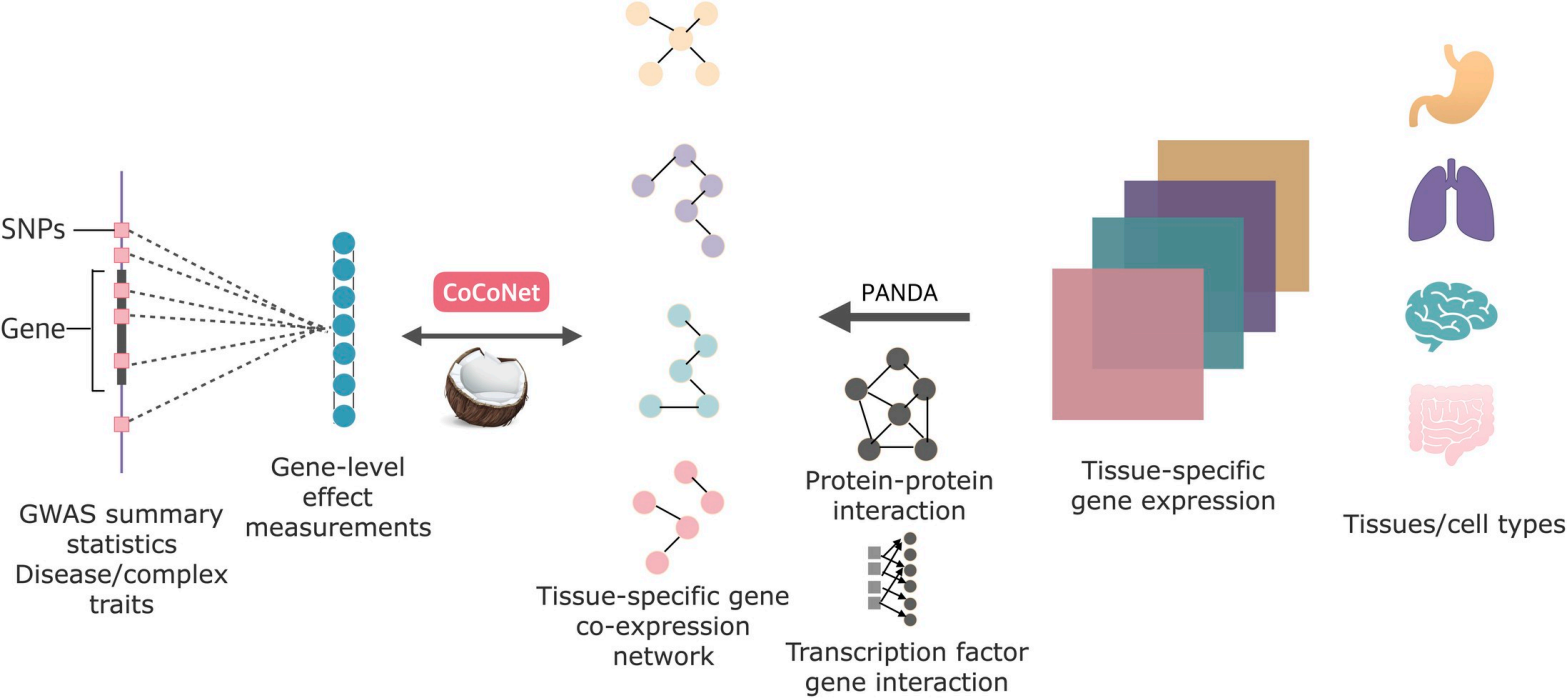
Schematic of CoCoNet for identifying trait relevant tissue/cell type using information from gene co-expression networks. From left to middle: we calculate gene level effect measurements from GWAS summary statistics for the trait of interest and treat them as an *m* dimensional vector of outcomes. From right to middle: we infer the tissue specific gene co-expression network for each tissue/cell type using the PANDA software, which requires the tissue-specific gene expression matrix, existing protein-protein interaction network information, as well as existing transcription factor and gene binding information as input. For the trait of interest, we examine one tissue at a time and we model the gene-level effect measurements for the trait as a function of the gene co-expression matrix using a covariance regression network model. We infer parameters in the model through composite likelihood. We calculate the maximum composite likelihood for each tissue and eventually rank tissues by the corresponding log likelihoods.

Our method is described in the Materials and Methods, with technical details provided in the S1 Text and a schematic showing in Fig 1. Briefly, our method requires both gene-level effect measurements obtained from GWAS on the trait of interest and tissue-specific gene co-expression adjacency matrices inferred from gene expression studies. For a given trait of interest, we examine one tissue at a time and model the gene-level effect measurements for the trait as a function of the gene co-expression matrix using a covariance regression network model. To ensure model robustness and scalable computation, we infer parameters in the covariance regression network model through composite likelihood. We calculate the maximum composite likelihood for each tissue and eventually rank tissues by the corresponding log likelihoods. We refer to our method as CoCoNet, freely available as an R software package.

We first examined the performance of our method through simulations (details in Materials and Methods). Briefly, we created tissue-specific gene co-expression adjacency matrices for 1,000 genes from 10 tissues in GTEx. We randomly selected one tissue as the true trait-relevant tissue and used its gene co-expression adjacency matrix to simulate the gene-level effect measurements for the trait. Afterwards, we examined one trait at a time using CoCoNet and calculated the power to detect the true trait-relevant tissue. We examined a total of three scenarios.

**Fig 2:**
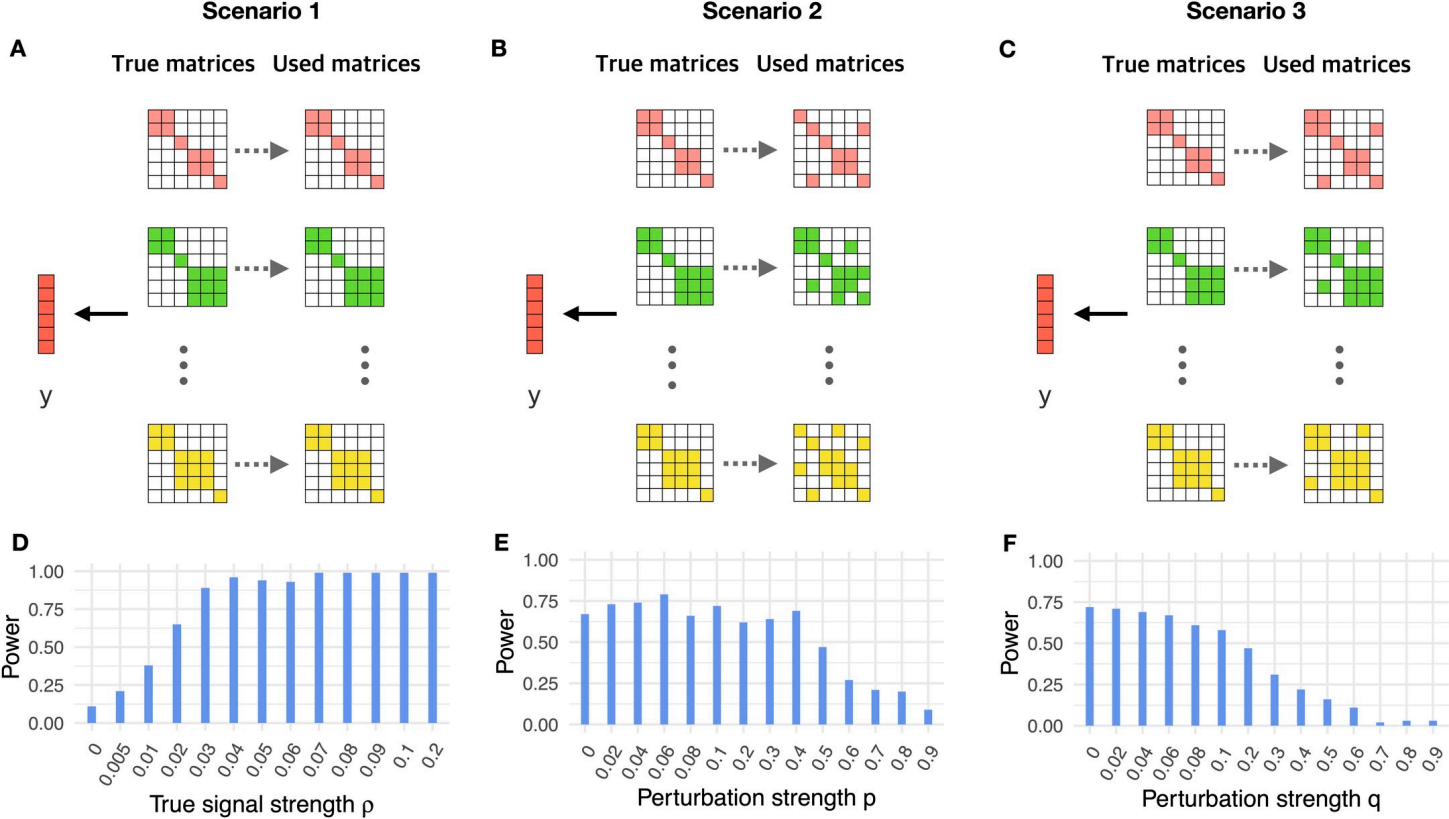
Power of CoCoNet to identify trait relevant tissues in simulations. (**A**): In scenario I, we selected one of the ten tissues as the trait-relevant tissue (second row) and simulated gene-level effect measurements based on the adjacency matrix (first row). We treated the adjacency matrices all as observed (third row) and fit the model using each of the ten matrices. (**B**): In scenario II, the data are generated in the same way as in scenario I but we fit the model using tissue-specific matrices that are noisy versions of the truth (third row). In particular, we assumed that each connected gene pair in the true adjacency matrices has a probability of *p* being un-observed, and each unconnected gene pair has a probability *q* to be falsely assigned as connected. (**C**): In scenario III, the data are again generated in the same way as in scenario I but we fit the model using tissue-specific matrices that are noisy versions of the truth (third row). In particular, we randomly converted a proportion *q* of unconnected gene pairs in the true adjacency matrix to be connected. (**D**): The power of CoCoNet for identifying the correct tissue (y-axis) increases with increasing signal strength measured by $\rho =\sigma_{1}^{2}/(\sigma_{1}^{2}+\sigma_{0}^{2})$ (x-axis) in scenario I. (**E**): The power of CoCoNet for identifying the correct tissue (y-axis) gradually decreases with increasing noise level characterized by *p* (x-axis) in scenario II. (**F**): The power of CoCoNet for identifying the correct tissue (y-axis) gradually decreases with increasing noise level characterized by *q* (x-axis) in scenario III.

In scenario I, we fit the data using the tissue-specific gene co-expression adjacency matrices. We varied the signal strength parameter *ρ* within the model to examine the estimation accuracy and power of our method across a range of signal strength (Fig 2A). We found that our composite likelihood-based method can estimate model parameters relatively well (S2A and S2D Fig, S3A and S3D Fig, S4A and S4D Fig). For example, when *ρ* = 0.02, which is close to the median estimate in the real data applications, the *ρ* estimates across 100 simulation replicates are centered around 0.017, with a standard deviation of 0.013. In addition, our method has descent power in detecting the true trait-relevant tissue across a range of *ρ* (Fig 2D). For example, when *ρ* = 0.02, the power of our method is 0.65. The power of our method also increases with increasing *ρ*. For example, the power of our method increases to 0.89 when *ρ* = 0.03.

In scenario II, when we fit the simulated data, we assumed that we did not observe the true tissue-specific gene co-expression adjacency matrices. Instead, we only observed a noisy version of them (Fig 2B). In particular, we added noise into each of the adjacency matrices by assuming that each pair of the connected genes has a probability of *p* being unobserved, and each pair of unconnected genes has a probability *q* to be falsely assigned as connected. For each fixed value *p*, we calculated *q* such that in expectation the proportion of connected gene pairs in the adjacency matrix equals to that of the original one. Here, we fixed *ρ* = 0.02 and varied *p* to examine its influence on parameter estimates and power. As expected, with increasing *p*, the observed adjacency matrix deviates further from the true networks. Subsequently the accuracy of the parameter estimates *ρ* (S2B and S2E Fig) and $\sigma_{1}^{2}$ (S4B and S4E Fig) reduces; though the $\sigma_{0}^{2}$ estimates are relatively stable and accurate (S3B and S3E Fig). Importantly, however, the power of our method in detecting the trait relevant tissue was relatively stable for small *p* (e.g. *p*<0.4), remaining around 0.7 (Fig 2E). Certainly, with increasingly large *p*, the power of our method gradually decreases towards the null expectation of 0.1.

In scenario III, we also assumed that the observed gene co-expression adjacency matrices are a noisy version of the truth (Fig 2C). Different from scenario II, however, we assumed here that the true adjacency matrices can be considered as a subset of the observed adjacency matrices, mimicking the setting where only the regulatory network of a set of core genes influences gene-level effect measurements on the trait of interest. In particular, we assigned noise to the networks by randomly converting unconnected gene pairs in the true adjacency matrices to be connected with a probability *q*. Here, we fixed *ρ* = 0.02 and varied the value of *q* to examine its influence on parameter estimation and power. As expected, because the noise added to the matrices makes it hard to perform accurate estimation, the accuracy of *ρ* (S2C and S2F Fig) and $\sigma_{1}^{2}$ (S4C and S4F Fig) become worse with increasing *q*; though the $\sigma_{0}^{2}$ estimates are relatively stable and accurate (S3C and S3F Fig). Importantly, however, the power of our method in detecting trait relevant tissues remains stable and is around 0.7 across a range of reasonable *q* (*q*<0.1); the power only starts to decrease with continuously increasing *q* (Fig 2F).

### Real data application: Inferring trait-relevant tissues with bulk RNAseq

We focus our first real data application on eight different disease GWASs that include four neurological disorders and four autoimmune disorders. We focus on these diseases because their disease relevant tissues have been reasonably well characterized and the corresponding GWASs have sufficiently large sample sizes (>12,000). Here, we aim to identify for each disease the trait-relevant tissue among 38 tissues obtained from GTEx. To do so, for each of the 38 tissues in turn, we followed Sonawane et al. [41] and used the PANDA software to infer a tissue-specific gene co-expression adjacency matrix. These inferred adjacency matrices contain tissue specific information (S5 Fig and S6 Fig). For example, the adjacency matrices for the three brain tissues (basal ganglia, cerebellum, and brain other) are all clustered together. Similarly, the adjacency matrices for the intestinal tissues (stomach, colon-transverse, intestine terminal ileum, and colon sigmoid) are all clustered together. With the adjacency matrices, we applied CoCoNet to examine one tissue at a time and ranked the relevance of tissue to disease by log likelihood.

**Fig 3:**
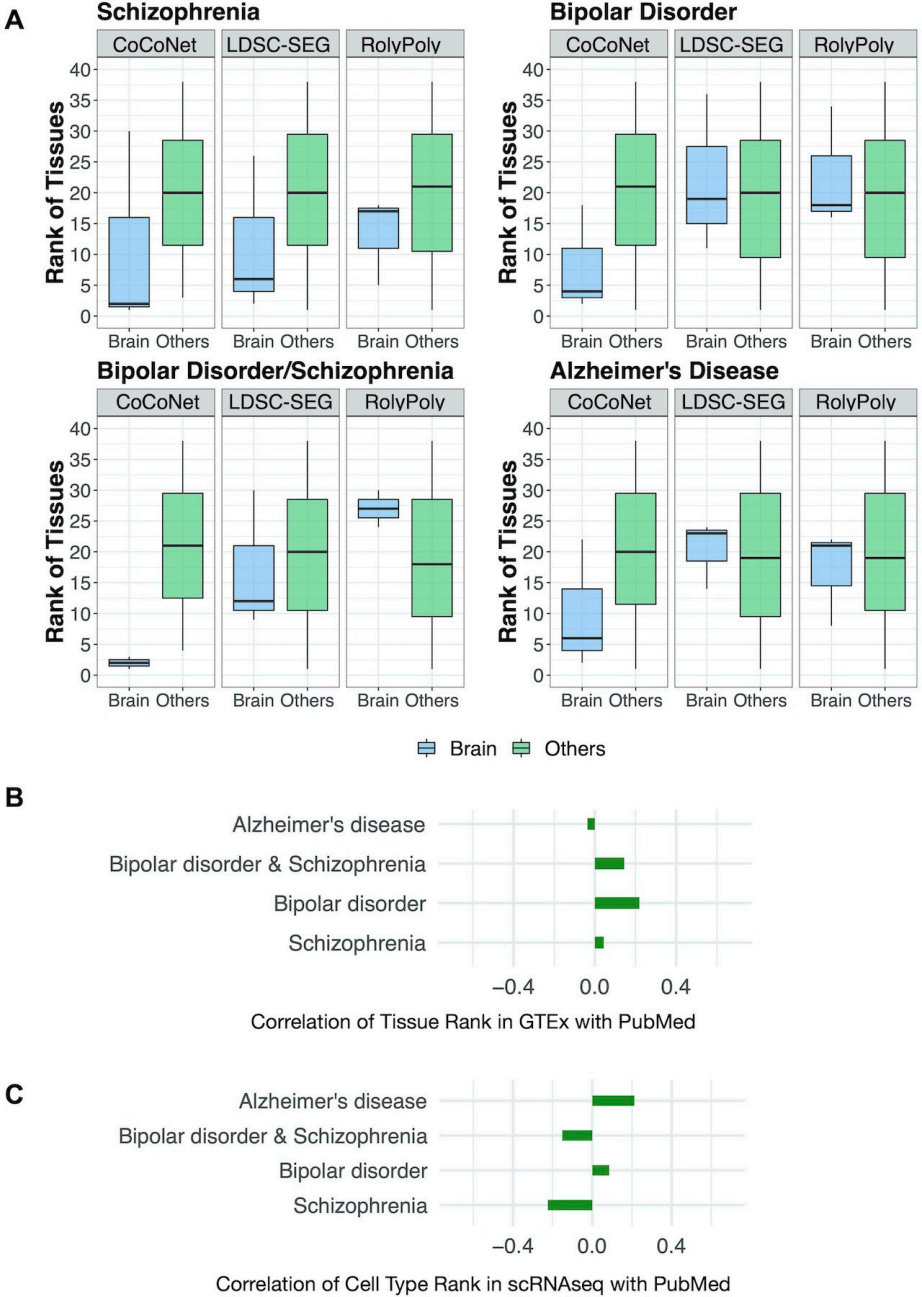
Comparison of tissue rankings by CoCoNet, LDSC-SEG, and RolyPoly for four neurological traits. (**A**): For each of the four neurological diseases, we plotted the rank (y-axis) of three brain tissues (colored blue; including brain cerebellum, brain basal ganglia, and brain other) and the rank of the remaining 35 tissues (colored green) in separate boxplots. The rank of brain tissues obtained using CoCoNet is often lower than that of the non-brain tissues (first column in each panel). The rank difference between the two types of tissues obtained by CoCoNet (first column in each panel) is often more pronounced than that obtained by either LDSC-SEG (second column) or RolyPoly (third column) for the four neurological diseases. (**B**): We compared the ranking of tissues obtained by CoCoNet with that obtained by PubMed search for 4 neurological traits, in the bulk RNAseq data. The CoCoNet and PubMed search results are positively correlated with each other for three traits. (**C**): We compared the ranking of cell types obtained by CoCoNet with that obtained by PubMed search for 4 neurological traits, in the single cell RNAseq data. The CoCoNet and PubMed search results are positively correlated with each other for two traits.

**Fig 4:**
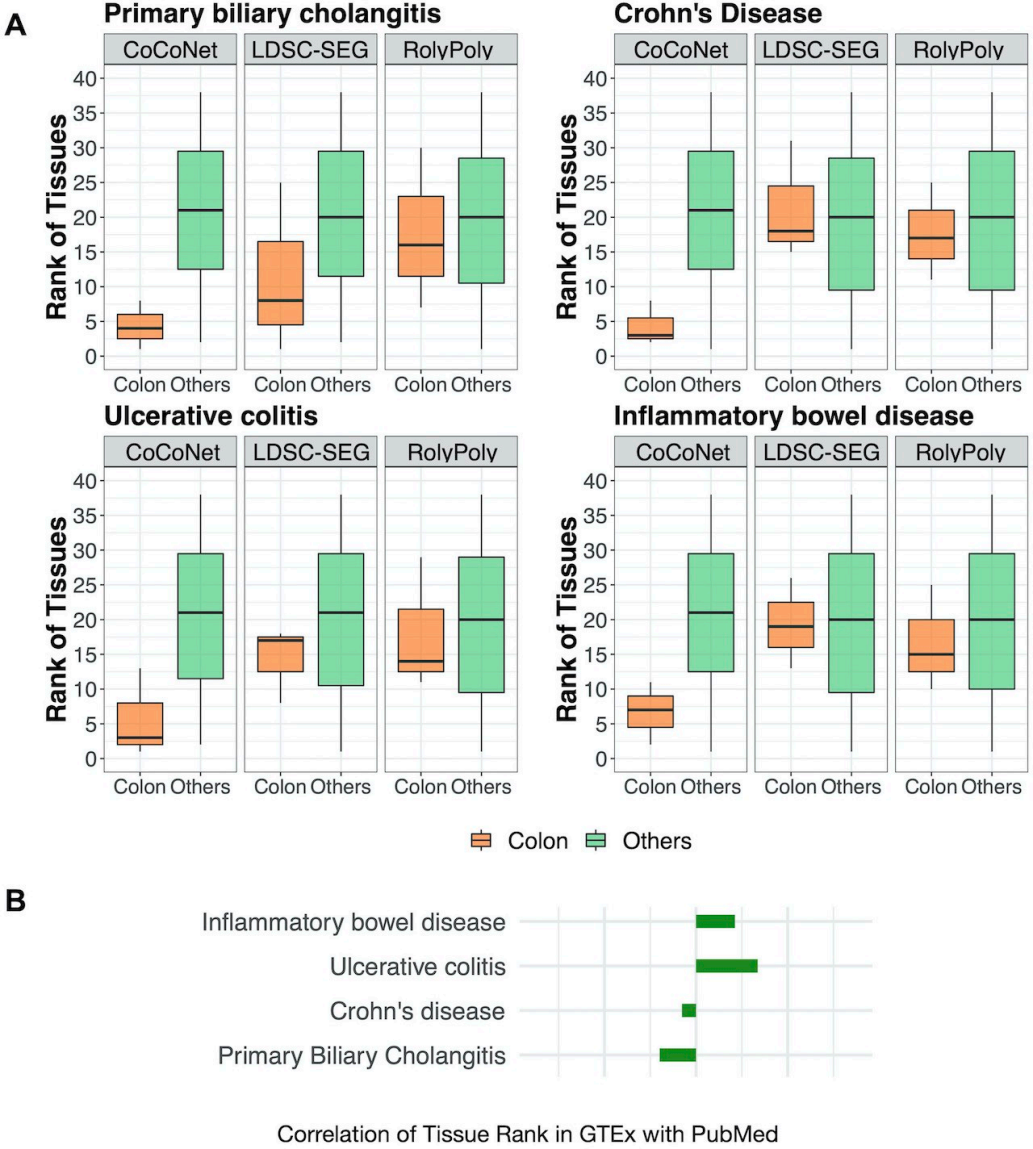
Comparison of tissue rankings by CoCoNet, LDSC-SEG, and RolyPoly for four autoimmune traits. (**A**): For each of the four autoimmune diseases, we plotted the rank (y-axis) of three colon tissues (colored orange; including colon sigmoid, colon transverse, and intestine terminal ileum) and the rank of the remaining 35 tissues (colored green) in separate boxplots. The rank of colon tissues obtained using CoCoNet is often lower than that of the non-colon tissues (first column in each panel). The rank difference between the two types of tissues obtained by CoCoNet (first column in each panel) is often more pronounced than that obtained by either LDSC-SEG (second column) or RolyPoly (third column) for the four autoimmune diseases. (**B**): We compared the ranking of tissues obtained by CoCoNet with that obtained by PubMed search for 4 autoimmune traits, in the bulk RNAseq data. The CoCoNet and PubMed search results are positively correlated with each other for two traits.

Overall, we found that the top relevant tissues identified for neurological disorders are generally brain tissues and the top relevant tissues identified for autoimmune disorders are generally intestinal tissues (Table 1 and S7 Fig). For example, at least one brain tissue is identified either as the most relevant or the second most relevant tissue for all four neurological disorders (Table 1 and S7A–S7D Fig). Similarly, at least one intestinal tissue is identified either as the most relevant or the second most relevant tissue for all four autoimmune disorders (Table 1 and S7E–S7H Fig). The inferred ranking of tissues for each disease does not depend on the sample size of each tissue. Specifically, Spearman’s rank correlation between tissue ranking and tissue sample size ranges from -0.32 to 0.13 for the eight diseases, with none of them being significant (S8 Fig). Besides CoCoNet, we analyzed the same data with RolyPoly and LDSC-SEG, both of which use tissue-specific gene expression information in place of tissue-specific gene co-expression network information for trait-tissue relevance inference. In the comparison, we found that the ranking of brain tissues for neurological disorders obtained by CoCoNet is higher than that obtained by RolyPoly or LDSC-SEG (Fig 3A). Similarly, the ranking of colon tissues for autoimmune disorders obtained by CoCoNet is also higher than that obtained by RolyPoly or LDSC-SEG (Fig 4A). The comparative results between CoCoNet and RolyPoly/LDSC-SEG suggests that tissue-specific gene co-expression network provides valuable trait-tissue relevance information, more so than the information provided by tissue-specific gene expression pattern used in RolyPoly or LDSC-SEG. Finally, we note that distance between pairs of genes may confound our analysis results. Specifically, if two genes are close to each other, then the gene-level effect measurements might be similar to each other. In addition, if two genes are close to each, they also tend to be co-regulated and thus co-expressed. Subsequently, the distance between genes could be an important confounder of our analysis. We indeed observed correlation between gene-effect measurement difference and distance for pairs of genes for all traits, though such correlation is not always in the expected positive direction (S9 Fig; p-value in the range of <2.2e-16 to 0.004 for the eight diseases). Nevertheless, we controlled for the gene pair-wise distance matrix as an additional term in CoCoNet (details in S1 Text). After controlling for distance confounding, the overall ranking of brain tissues and intestinal tissues does appear to improve for most traits (S10 Fig vs S7 Fig).

**Table 1:** Top five tissue types identified among the 38 tissues in the GTEx bulk RNAseq data for each of the eight GWAS traits.

Traits	Top1	Top2	Top3	Top4	Top5
Schizophrenia	Brain basal ganglia	Brain other	Uterus	Skin	Tibial Nerve
Bipolar Disorder	Prostate	Brain basal ganglia	Esophagus mucosa	Breast	Brain cerebellum
Bipolar Disorder/ Schizophrenia	Brain basal ganglia	Brain cerebellum	Spleen	Brain other	Thyroid
Alzheimer’s Disease	Testis	Brain other	Colon sigmoid	Colon transverse	Intestine terminal ileum
Primary biliary cholangitis	Intestine terminal ileum	Esophagus mucosa	Colon sigmoid	Stomach	Artery coronary
Crohn’s Disease	Testis	Colon transverse	Artery coronary	Colon sigmoid	Heart atrial appendage
Ulcerative colitis	Stomach	Whole blood	Colon sigmoid	Fibroblast cell line	Breast
Inflammatory bowel disease	Testis	Colon sigmoid	Heart atrial appendage	Skeletal muscle	Tibial Nerve

We also attempted to validate the identified trait-relevant tissue by performing a PubMed search following the main idea in [8]. Specifically, we reasoned that, if a tissue is relevant to the disease of interest, then there would be previous publications studying the disease on the particular tissue. Therefore, by counting the number of previous publications using the key word pairs of trait and tissue, we would have a reasonable quantitative estimate on the relevance between the trait and the corresponding tissue, which can serve to validate the results obtained by our method. Here, we followed such intuition and counted the number of previous publications on pairs of trait and tissue (details in Materials and Methods). For each trait in turn, we ranked tissues by the number of previous publications on the trait-tissue pair. We then calculated the Spearman’s correlations between the tissue rank obtained by CoCoNet and the tissue rank obtained by PubMed search (Fig 3B and Fig 4B). Overall, the tissue ranking results obtained by CoCoNet is reasonably consistent with PubMed search results for majority of traits: the Spearman’s rank correlation between these two approaches is 0.07 on average (median = 0.09), with ranges from -0.15 (for PBC) to 0.26 (for UC); the correlation is positive for five out of eight traits. The correlation is lower than those obtained using histone modification marks (median = 0.417 based on [8]), consistent with the previous observation that histone modification contains more information than gene expression data for trait-relevant tissue inference [19].

As a concrete example, for schizophrenia, our method identified the basal ganglia as the most relevant tissue, which is consistent with PubMed search results. Basal ganglia is a functional brain region involved in a variety of activities including motor movements, cognition and emotion. Basal ganglia contains most of the dopamine neurons in the brain and dopamine is the first neurotransmitter implicated for schizophrenia [47]. Indeed, the dopaminergic system of the basal ganglia is known to display several anomalies in schizophrenia and is itself a target of various antipsychotic drugs [47]. As another example, for Crohn’s disease, our method identified colon transverse and colon sigmoid as the most relevant tissues. The results are consistent with PubMed search results, which identified colon transverse and colon sigmoid as the most relevant tissues. Crohn’s disease is a chronic inflammatory disease that is known to affect all segments of the gastrointestinal tract, with particular pathological influence on the terminal ileum and colon [48].

### Real data application: Inferring trait-relevant cell types with single cell RNAseq

Our second application focuses on GWASs of the four neurological diseases described in the previous section. For each disease in turn, we aimed to identify the trait-relevant cell types. To do so, we constructed gene co-expression adjacency matrices for each of the 10 cell types from two donors using single cell sequencing data [44]. The 10 cell types include five neuronal cell types, four glial cell types, and one endothelial cell type. For each cell type in turn, we inferred a gene co-expression adjacency matrix through the same analytic pipeline described in the previous section. Note that PANDA is designed specifically for bulk RNAseq data but its utility has not been shown for single cell RNAseq data. Therefore, we caution the over-interpretation of the results presented here. Nevertheless these inferred gene co-expression adjacency matrices contain cell type specific information (S11 Fig and S12 Fig). For example, astrocytes from two donors were clustered together, along with other glia cells such as microglia, oligodendrocytes, and oligodendrocyte precursor cells. The neuronal cells, such as glutamatergic neurons, GABAergic interneurons, pyramidal neurons, and granule neurons were also clustered together. Therefore, in our following analysis, we combined the adjacency matrices for the same cell type from the two donors into a single adjacency matrix through union operation. For each disease in turn, we applied our method to examine one cell type at a time and ranked the relevance of cell types to the disease by log likelihood (Table 2, S13 Fig). The ranking of cell types for each neurological disease does not depend on the number of cells in each cell type (Spearman’s rank correlation ranges from -0.39 to 0.33; only one statistically significant) (S14 Fig). We also compared the cell ranking results obtained by CoCoNet to those obtained through PubMed search (Fig 3C). Likely due to high experimental noise in single cell RNAseq data, the cell ranking results obtained by CoCoNet is consistent with PubMed search results for two out of four diseases: the rank correlation is 0.21 for Alzheimer’s disease and 0.08 for Bipolar disorder. Finally, we also observed correlation between gene-effect measurement difference and distance difference for pairs of genes, with correlation direction varied across traits (S15 Fig). After controlling for distance confounding, the overall rankings of glias, neurons and other cell types does not appear to change much for most traits (S16 Fig).

**Table 2:** Top five cell types identified among 10 cell types in the single cell RNAseq data for each of the four neurological disorders.

Traits	Top1	Top2	Top3	Top4	Top5
Schizophrenia	Granule neurons	Microglia	Endothelial cell	Oligodendrocyte precursor cells	Pyramidal neurons
Bipolar Disorder	Pyramidal neurons	Microglia	Oligodendrocyte precursor cells	Oligodendrocyte	Astrocytes
Bipolar Disorder/ Schizophrenia	Oligodendrocytes	Endothelial cell	Glutamatergic neurons	Astrocytes	Granule neurons
Alzheimer’s Disease	GABAergic interneurons	Oligodendrocyte precursor cells	Astrocytes	Microglia	Endothelial cell

Cell types identified in the top trait-cell type relevance list for these neurological diseases often make biological sense (S13 Fig). For example, for Alzheimer’s disease, we ranked GABAergic interneurons, oligodendrocyte precursor cells, astrocytes, and microglia as top relevant cell types. Both astrocytes and microglia were identified by PubMed search as the most relevant cell types. These two glia cell types are activated by the pathological peptide amyloid beta and release proinflammatory mediators to induce neuronal death [49, 50]. Similarly, significant reductions in GABA levels and reduced GABAergic innervations are commonly observed during the progression of Alzheimer’s disease. Such GABA reduction leads to eventual disruption of the neuronal circuitry and impaired cognition [51]. Finally, vulnerability of oligodendrocyte precursor cells under Alzheimer's pathology can induce myelin breakdown and loss of myelin sheath, which characterizes the earliest stage of Alzheimer’s disease [52]. As another example, for bipolar disorder, both pyramidal neurons and various glia cells are selected as top relevant cell types. In patients with bipolar disorder, the size of pyramidal neurons in the CA1 region of the hippocampus are often decreased, suggesting pathogenic effects of bipolar disorder on pyramidal neurons [53]. Similarly, it has been shown that decreased density of glial cells may contribute to the pathological changes observed in neurons in bipolar disorder patients [54].

## Discussion

We have presented a new method to leverage gene co-expression patterns for inferring trait-relevant tissue or cell types. With the real data examples, we show that tissue-specific gene co-expression patterns contain valuable information for inferring trait-tissue relevance.

There are two important ingredients in our method. The first key ingredient is the tissue-specific gene co-expression matrix. In the present study, we have primarily used the adjacency matrix based on hard thresholding the tissue-specific gene co-expression matrices obtained from [41]. Because of the small sample sizes relative to the number of genes in the gene expression study, matrix sparsity introduced by hard thresholding is crucial for improving the signal contained in the co-expression matrices. While hard thresholding is one of the easiest approaches to introduce matrix sparsity, other sophisticated sparse matrix methods, such as graphical lasso [55, 56], may have added benefits and are worth future exploration. In addition, we have primarily focused on using the adjacency matrix directly in the covariance function. The covariance function used in CoCoNet can be general and can consist of higher order terms of the adjacency matrix through the polynomial matrix construction. We have implemented our composite likelihood method to accommodate high order terms of the adjacency matrix as determined by the parameter *K*. However, we found that models with larger *K* tend to have higher BIC in the two data applications we examined here, indicating that direct connections between genes may contain sufficient information for trait tissue relevance inference. Besides the use of polynomial matrix construction, alternative ways to construct the covariance function, such as the use of graph Laplacian matrix [57], could be an interesting area for future exploration.

We note that our adjacency matrices are constructed based on the PANDA software. The PANDA software can integrate multiple data sources to facilitate the construction of tissue-specific gene co-expression matrices and are thus commonly used previously (e.g. it was used to construct the tissue specific co-expression matrices in GTEx [41]). Here, we focused the gene co-expression matrices only on genes (i.e. nodes) and gene pairs (i.e. edges) that are specific in tissues. We also transformed the co-expression matrices output by PANDA to adjacency matrices by using a user-specified hard threshold. We varied such hard threshold and found the trait-tissue relevant results are relatively robust regardless of what hard thresholds we used (S17 Fig). However, consistent with [18, 19], we did notice that it is important to focus on genes and gene pairs that are specific in at least one tissues; otherwise the results become insensible (S18 and S19 Figs). Importantly, PANDA uses multiple data sources that include gene expression data, protein-protein interaction, as well as transcription factor binding information. Besides the default PANDA data sources, there are many other software and informative databases for constructing gene-gene co-expression networks. For example, GIANT (Genome-scale Integrated Analysis of gene Networks in Tissues) is a tissue-specific interaction network database that integrates physical interaction, co-expression, miRNA binding motif and transcription factor binding site data [2]. Such a database can be paired with PANDA to facilitate accurate gene co-expression network construction. Besides PANDA, various other methods for inferring tissue-specific gene co-expression patterns can also be used in pair with CoCoNet. Some of these methods, such as ARACNE (Algorithm for the Reconstruction of Accurate Cellular Networks) [58] and WGCNA (weighted gene co-expression network analysis) [43], only rely on gene expression data. Some of these methods, such as GRAM (Genetic Regulatory Modules) [59], CLR (Context likelihood of relatedness) [60] and GENIE3 (GEne Network Inference with Ensemble of trees) [61], can integrate gene expression data together with transcription factor information; though none of these methods can incorporate protein-protein interaction information as PANDA does. Here, we briefly explored the use of WGCNA to construct the tissue-specific adjacency matrices and found the results to be similar but less (S20 Fig). Nevertheless, exploring the benefits of different methods in constructing tissue-specific gene co-expressing pattern for trait-tissue relevance inference remains an important future area of research. Finally, while we have explored the use of PANDA for a single cell/nucleus RNAseq data, we fully acknowledge that PANDA was not designed specifically for constructing gene co-expression network from single cell RNAseq data. In particular, specific features of single cell data such as extremely low counts and excessive dropout events may influence the effectiveness of PANDA or even its validity. Therefore, we caution the over-interpretation of the single cell results and emphasize that use of methods that are specifically designed for constructing cell type specific gene co-expression network from single cell RNAseq data [62–66] is an important future direction.

The second key ingredient of our method is the gene-level effect measurements for the GWAS trait. When the gene-level effect measurements are ordered quantitative statistics either without signs or with signs that are not biologically meaningful (i.e. positively signed value is higher than the negatively signed value and the negative sign does not represent negative effect), it seems nature to make the modeling assumption in CoCoNet that if two genes share similar functionality then the two genes will likely have similar effects on the trait of interest. When the gene-level effect measurements are signed with signs being biologically meaningful (i.e. negative sign represents negative effect), then the CoCoNet modeling assumption is no longer valid. For example, when two genes belong to the same pathway and have opposite effects, then the signed gene-level effects of the two genes are no longer correlated with each other even though the two genes are connected with each other in the gene co-expression network. In this case, we can use the transform the signed gene-level effects into its unsigned version by taking the absolute value. In addition, when binary gene-level effect measured are provided, we can also make use of the Ising model in place of the covariance regression model in CoCoNet. The Ising model effectively assumes that two genes with similar functions will have the same categorization of the outcome which can be a useful modeling assumption in certain scenarios (S1 Text). In the present study, we have primarily used gene-level per-SNP heritability estimates as the unsigned gene-level effect measurements. The gene-level heritability not only has a natural genetic interpretation but is also directly linked to the gene-level effect measurement in the sequence kernel association test (SKAT) [67], which has been widely used for SNP set tests. Besides gene-level heritability, we have also explored the use of the test statistics for gene-level heritability, which is closely related to the SKAT test statistics as the outcome variable in CoCoNet (S21 Fig) [8]. The test statistics for gene-level heritability is defined as the per-SNP gene-level heritability divided by its standard error. Results based on the gene-level heritability test statistics are largely consistent with our main analysis though with noticeable weaker performance (e.g. S21 Fig vs S7 Fig). In particular, brain tissues remain highly ranked for three out of four neurological diseases (instead of all four in the main analysis) and the colon tissues remain highly ranked for two out of four immunological diseases (instead of all four in the main analysis). The results suggest that the gene-level effect measurement is an important contributor that influence the power of CoCoNet. Besides the test statistics for gene-level heritability, many SNP set methods exist for carrying out gene-level tests. Common SNP set methods include the versatile gene-based test (VEGAS)[68], the MinP approach [69] standard combination statistics (e.g. Fisher, Sidat, and Simes) [70], and the principal component analysis regressions [71]. Different SNP set methods have different benefits and disadvantages, and consequently, gene-level effect measurements constructed from different methods may contain different levels of information for inferring trait-tissue relevance. CoCoNet can be paired with gene-level statistics constructed from any SNP set test and it would be important to explore the benefits of different gene-level statistics for trait-tissue relevance inference in the future.

Note that we have only focused on ranking tissues for a given disease instead of directly testing the tissue relevance to the disease. Ranking tissues requires model selection, which is an easier analysis task than carrying out hypothesis testing for tissue relevance to disease. Hypothesis testing on the tissue relevance to disease using gene co-expression patterns is challenging because of the strong correlation among gene co-expression patterns constructed from different tissues. Specifically, the gene co-expression matrices constructed in different tissues are highly correlated with each other. Because of the high correlation across gene co-expression matrices, the estimated maximum likelihood across different tissues using CoCoNet can be similar to each other. Subsequently, the naïve *p-*value for testing trait-tissue relevance would become significant even for trait-irrelevant tissues, simply due to tissue-tissue correlation. Therefore, a naïve hypothesis test would lead to an undesirably large number of false positives. Previous studies using gene-level epigenomic annotations for trait-tissue relevance inference have also encountered a similar phenomenon. Indeed, previous studies have to either introduce a large set of covariates to account for tissue-tissue correlation [15], or rely on a mixture model to formulate the hypothesis test into a model selection framework [8]. Unfortunately, we found that adjusting for known gene-level covariates is not effective for addressing correlation among gene co-expression matrices across tissues. In addition, a mixture model-based model selection approach is technically challenging in our setting due to the small difference in the estimated maximum likelihood across tissues. Therefore, we have resorted to the easier task of ranking tissues for a given disease and focus on the top selected tissues for biological interpretation. To quantify the uncertainty in tissue ranking, we performed subsampling on each tissue specific adjacency matrix and asked how consistent the rank of a given tissue is between the original data and the subsampled data. In particular, we computed a reproducibility score, which provides an uncertainty quantification of tissue ranking (details in S1 Text). As expected, we found that the reproducibility score reduces with increasing rank in the simulations (S22 Fig), supporting that high rank is often associated with low uncertainty. In the real data, reproducibility scores for top ranks are often reasonably high (S6 Table and S23 Fig), with consistency in tissue rank between the original data and subsampled data varying across traits (S24 Fig). Despite the apparent effectiveness of the reproducibility score, however, we acknowledge that such score remains somewhat *ad hoc* in nature. Therefore, future methodological innovations are needed to directly infer statistical confidence in the ranking itself or to develop effective hypothesis tests to incorporate gene co-expression pattern for trait-relevant tissue inference.

Finally, we would like to acknowledge that, while the gene co-expression network appears to contain positive information for trait-tissue relevance inference, such information is not over-whelming as revealed in the present study. For example, our PubMed correlation results show that the tissue ranking is largely consistent with previous research publications only for some tissues but not for all. In addition, the PubMed correlation results suggest that the gene co-expression information contains much lower information than that contained in histone modifications that previously reported [8] (median correlation value of 0.09 by using tissue-specific gene co-expression vs median correlation value of 0.417 by using tissue-specific histone modifications). Many negative results exist. For example, the top relevant tissues for autoimmune diseases are inferred by CoCoNet to be disease-target tissues that are in the digestive system, but not immune-related tissues (e.g. blood) that may directly cause the diseases. Therefore, trait-tissue relevance inferred by CoCoNet does not imply causation but merely association. In addition, “testis” pops up at the top one tissue for several diseases that does not appear to be sensible. Indeed, the lack of over-whelming information in gene co-expression network is also observed in other complex trait such as height, BMI, waist hip ratio, CAD (S25 and S26 Figs). For example, adipose subcutaneous is ranked 1^st^ in waist hip ratio and artery coronary is ranked 4^th^ in coronary artery disease, but no heart related tissues are ranked high for CAD. It is possible that the gene co-expression network inferred from the gene expression study is inaccurate due to the relatively small sample size in the current expression studies. It is also possible that CoCoNet is not yet optimized to extract and take advantage of the tissue specific gene co-expression information accurately and in a maximized fashion. Certainly, similar negative results are frequently observed in existing literature on trait-relevance inference [72–74]. For example, lung is inferred as the top tissue for autoimmune disease UC while lymphoblastoid cell line is inferred as the top tissue for neurological disease BIP in the original LDSC-SEG paper when we only focus on GTEx tissues. Similar, for schizophrenia, the top relevant tissue inferred by LDSC-SEG is pancreas while the top relevant tissue by RolyPoly is lymphoblastoid cell line in the present study. These negative results highlight the low statistical power and important statistical challenges of using gene expression data alone, especially using only gene co-expression network information as in CoCoNet, for trait-relevant tissue inference.

## Supporting information

S1 Fig A CoCoNet model with a small *K* is often preferred than a CoCoNet model with a large *K* in real data sets. Here, *K* is the number of matrices included in the covariance function. In the real data application, we analyzed all pairs of 38 GTEx tissues and 8 GWAS traits. For each of the 304 trait-tissue pairs, we fit four different CoCoNet models with K ranging from 1 to 4. The above histogram shows the Bayesian information criterion (BIC) values (x-axis) across all these CoCoNet models. Models with different *K* are colored differently: A1 represents a model with 1^st^ power of the adjacency matrix (red); A2 represents a model with both 1^st^ and 2^nd^ power of the adjacency matrix (green); A3 represents a model with up to the 3^rd^ power of the adjacency matrix (blue); A4 represents a model with up to the 4^th^ power of the adjacency matrix (purple). The results suggest that a model with a low value of *K* (1 or 2) often comes with a lower BIC and is thus often preferred than a model with a high *K*.
(TIF)

S2 Fig Parameter estimation of the signal strength parameter *ρ* in simulations. (A): Boxplot shows the ρ estimates across 100 simulation replicates for each true ρ (x-axis) in the simulation scenario I. (B): Boxplot shows the ρ estimates across 100 simulation replicates for each parameter p (x-axis) in the simulation scenario II. Here, true ρ = 0.02. Increasing parameter p adds increasingly large noise to the tissue-specific adjacency matrices, thus leading to downward biased estimation of ρ. (C): Boxplot shows the ρ estimates across 100 simulation replicates for each parameter q (x-axis) in the simulation scenario III. Here, true ρ = 0.02. Increasing parameter q adds increasingly more noise to the tissue-specific adjacency matrices, thus leading to downward biased estimation of ρ. (D-F): mean squared error (MSE; y-axis) measures the accuracy of ρ estimates across the three simulation scenarios.
(TIF)

S3 FigParameter estimation of the parameter $\sigma_{0}^{2}$ in simulations.(A): Boxplot shows the $\sigma_{0}^{2}$ estimates across 100 simulation replicates for each signal strength parameter ρ (x-axis) in the simulation scenario I. (B): Boxplot shows the $\sigma_{0}^{2}$ estimates across 100 simulation replicates for each parameter p (x-axis) in the simulation scenario II. Here, true $\sigma_{0}^{2}=0.98$. Increasing parameter p adds increasingly large noise to the tissue-specific adjacency matrices, but does not appear to strongly influence the estimation of $\sigma_{0}^{2}$. (C): Boxplot shows the $\sigma_{0}^{2}$ estimates across 100 simulation replicates for each parameter q (x-axis) in the simulation scenario III. Here, true $\sigma_{0}^{2}=0.98$. Increasing parameter q adds increasingly large noise to the tissue-specific adjacency matrices, but does not appear to strongly influence the estimation of $\sigma_{0}^{2}$. (D-F): mean squared error (MSE; y-axis) measures the accuracy of $\sigma_{0}^{2}$ estimates across the three simulation scenarios.(TIF)

S4 FigParameter estimation of the parameter $\sigma_{1}^{2}$ in simulations.(A): Boxplot shows the $\sigma_{1}^{2}$ estimates across 100 simulation replicates for each signal strength parameter ρ (x-axis) in the simulation scenario I. (B): Boxplot shows the $\sigma_{1}^{2}$ estimates across 100 simulation replicates for each parameter p (x-axis) in the simulation scenario II. Here, true $\sigma_{1}^{2}=0.02$. Increasing parameter p adds increasingly large noise to the tissue-specific adjacency matrices, but does not appear to strongly influence the estimation of $\sigma_{1}^{2}$. (C): Boxplot shows the $\sigma_{1}^{2}$ estimates across 100 simulation replicates for each parameter q (x-axis) in the simulation scenario III. Here, true $\sigma_{1}^{2}=0.02$. Increasing parameter q adds increasingly large noise to the tissue-specific adjacency matrices, but does not appear to strongly influence the estimation of $\sigma_{1}^{2}$. (D-F): mean squared error (MSE; y-axis) measures the accuracy of $\sigma_{1}^{2}$ estimates across the three simulation scenarios.(TIF)

S5 Fig Adjacency matrices constructed from 38 tissues in the GTEx RNAseq data display tissue specific information. Jaccard index is computed between pairs of matrices to measure the similarity among adjacency matrices across tissues. Adjacency matrices on similar tissues tend to cluster together based on hierarchical clustering. For example, the adjacency matrices for the three brain tissues (green), such as basal ganglia, cerebellum, and brain other, are all clustered together. Similarly, intestinal tissues (red), such as stomach, colon-transverse, intestine terminal ileum, and colon sigmoid, are all clustered together.
(TIF)

S6 Fig Gene connectivity in the adjacency matrices constructed from 38 tissues in the GTEx RNAseq data displays tissue specific information. In each tissue, we calculated for each gene a node connectivity value, which measures the number of genes it is directly connected to a gene with a high node connectivity value is often referred to as a hub gene. Node connectivity values are similar between similar tissues as measured by Pearson’s correlation; thus similar tissues tend to cluster together based on Pearson’s correlation.
(TIF)

S7 Fig Rank of 38 tissues in the GTEx data in terms of their relevance to each of the eight GWAS traits obtained using CoCoNet. For each GWAS trait, we calculated the composite likelihood for each tissue, subtracted the minimum likelihood across all tissues (x-axis), and ranked traits based on these values from top to bottom in each panel (y-axis). The brain tissues are colored in blue; the colon related tissues are colored in yellow; and the rest of the tissues are colored in green. Brain tissues tend to rank high for the four neurological diseases (**A-D**) while colon related tissues tend to rank high for autoimmune diseases (**E-H**).
(TIF)

S8 Fig Tissue rank does not depend on tissue sample size for GWAS traits. The sample size of each tissue (y-axis) is plotted against the rank of each tissue by CoCoNet (x-axis) across eight GWAS traits (eight panels). Spearman’s rank correlation (R) between the tissue rank and the sample size, together with the corresponding *p*-value, are also displayed on the panels for neurological diseases (**A-D**) and autoimmune diseases (**E-H**). The tissue rank obtained by CoCoNet is not correlated with tissue sample size for all traits.
(TIF)

S9 Fig Difference in effect measurement is correlated with distance for pairs of genes across eight GWAS traits. Difference in effect **measurement** between two genes in a pair is measured by the absolute difference in per-SNP gene-level heritability (y-axis). Distance between two genes in a pair is measured by their TSS locations (x-axis). The Pearson’s correlation between the gene distance differences and the absolute gene effect measurements difference is calculated for neurological diseases (**A-D**) and autoimmune diseases (**E-H**).
(TIF)

S10 Fig Rank of 38 tissues in the GTEx data in terms of their relevance to four additional GWAS traits obtained using CoCoNet after controlling for gene distance. Here, based on the analysis in the main text, we use the distance matrix for genes as covariance. The distances between genes are measured by differences in transcript starting site (TSS) locations, then scaled by the maximum distance between genes. The distances between genes on different chromosomes are defined as 1 in the scaled distance matrix. For neurological diseases (**A-D**) and autoimmune diseases (**E-H**), we calculated the composite likelihood for each tissue, subtracted the minimum likelihood across all tissues (x-axis), and ranked traits based on these values from top to bottom in each panel (y-axis). The brain tissues are colored in blue; the intestinal related tissues are colored in yellow; and the rest of the tissues are colored in green. The results are highly consistent with the results in main text.
(TIF)

S11 Fig Heatmap showing Jaccard index among adjacency matrices constructed from multiple cell types in the single cell RNAseq data. Jaccard index between adjacency matrices constructed either from cell types in two donors separately (**A**) or from cell types merged from two donors (**B**) is measured by Jaccard index. Adjacency matrices on similar tissues tend to cluster together based on hierarchical clustering. For example, the same cell type from different donors tend to cluster together (**A**) and different glia cell types tend to cluster together (**B**). The Jaccard index between two identical matrices is 1, as shown on the diagonal with red color in the heatmap.
(TIF)

S12 Fig Gene connectivity in the adjacency matrices constructed from multiple cell types in the single cell RNAseq data. We calculated for each gene a node connectivity value, which measures the number of genes it is directly connected to a gene with a high node connectivity value is often referred to as a hub gene. Similarity between cell types constructed either from two donors separately (**A**) or from cell types merged from two donors (**B**) is measured by Pearson’s correlation. The Pearson’s correlation between two identical matrices is 1, as shown on the diagonal with red color in the heatmap.
(TIF)

S13 Fig Cell type rank does not depend on the number of cells in each cell type for GWAS traits. The number of cells in each tissue (y-axis) is plotted against the rank of each cell type by CoCoNet (x-axis) across neurological diseases (**A-D**). Spearman’s rank correlation (R) between the cell type rank and the number of cells, together with the corresponding *p*-value, are also displayed on the panels. The cell type rank obtained by CoCoNet is not correlated with the number of cells in the cell type for all traits.
(TIF)

S14 Fig Rank of 10 cell types in terms of their relevance for each of the four neurological diseases obtained through CoCoNet. For each of the four GWAS trait (**A-D**), we calculated the composite likelihood for each cell type, subtracted the minimum likelihood across all cell types (x-axis), and ranked cell types based on these values from top to bottom in each panel (y-axis).
(TIF)

S15 Fig Difference in effect measurement is correlated with distance for pairs of genes across four GWAS traits in single cell dataset application. Difference in effect measurement between two genes in a pair is measured by the absolute difference in per-SNP gene-level heritability (y-axis). Distance between two genes in a pair is measured by their TSS locations (x-axis). The Pearson’s correlation between the gene distance differences and the absolute gene effect measurements difference is calculated for each GWAS trait (**A-D**).
(TIF)

S16 Fig Rank of 10 cell types in terms of their relevance for each of the four neurological diseases after controlling for gene distance. Here, we use the distance matrix for genes as covariance. The distances between genes are measured by differences in transcript starting site (TSS) locations, then scaled by the maximum distance between genes. The distances between genes on different chromosomes are defined as 1 in the scaled distance matrix. For each of the four GWAS trait (**A-D**), we calculated the composite likelihood for each cell type, subtracted the minimum likelihood across all cell types (x-axis), and ranked cell types based on these values from top to bottom in each panel (y-axis).
(TIF)

S17 Fig Comparison of tissue rankings by different cut-off thresholds of edges in eight GWAS traits. In our main analysis, we performed a hard thresholding procedure to convert the continuous edge values to binary values. In particular, edges that had a positive edge value and that were specific to at least one tissue type were converted to one; otherwise, they were converted to zero. Here, we varied such hard threshold and found the trait-tissue relevant results are relatively robust regardless of what hard thresholds we used. (**A-D**): For each of the four neurological diseases, we plotted the rank (y-axis) of three brain tissues (colored blue; including brain cerebellum, brain basal ganglia, and brain other) and the rank of the remaining 35 tissues (colored green) in separate boxplots. (**E-H**): For each of the four autoimmune diseases, we plotted the rank (y-axis) of four intestinal tissues (colored orange; including colon sigmoid, colon transverse, and intestine terminal ileum, stomach) and the rank of the remaining 34 tissues (colored green) in separate boxplots.
(TIF)

S18 Fig Rank of 38 tissues in the GTEx data in terms of their relevance to each of the eight GWAS traits obtained using CoCoNet. Here, based on the analysis in the main text, we filtered the edges using same procedure, but used genes that are not specific in any of the 38 tissues, and also have mean gene expression greater than 10 to build the gene co-expression network. For neurological diseases (**A-D**) and autoimmune diseases (**E-H**), we calculated the composite likelihood for each tissue, subtracted the minimum likelihood across all tissues (x-axis), and ranked traits based on these values from top to bottom in each panel (y-axis). The brain tissues are colored in blue; the colon related tissues are colored in yellow; and the rest of the tissues are colored in green. The result indicates it is important to focus on genes that are specific in at least one tissues; otherwise the results become insensible.
(TIF)

S19 Fig Rank of 38 tissues in the GTEx data in terms of their relevance to each of the eight GWAS traits obtained using CoCoNet. Here based on the analysis in the main text, we build the networks using the edges that are non-specific in any tissue. For neurological diseases (**A-D**) and autoimmune diseases (**E-H**), we calculated the composite likelihood for each tissue, subtracted the minimum likelihood across all tissues (x-axis), and ranked traits based on these values from top to bottom in each panel (y-axis). The brain tissues are colored in blue; the colon related tissues are colored in yellow; and the rest of the tissues are colored in green. The result indicates it is important to focus on gene pairs that are specific in at least one tissues; otherwise the results become insensible.
(TIF)

S20 Fig Rank of 38 tissues in the GTEx data in terms of their relevance to each of the eight GWAS traits obtained using CoCoNet. Here, based on the analysis in the manuscript, we build the tissue specific networks through WGCNA only using gene expression data. For neurological diseases (**A-D**) and autoimmune diseases (**E-H**), we calculated the composite likelihood for each tissue, subtracted the minimum likelihood across all tissues (x-axis), and ranked traits based on these values from top to bottom in each panel (y-axis). The brain tissues are colored in blue; the colon related tissues are colored in yellow; and the rest of the tissues are colored in green. The result is relatively weak compared to using networks built in PANDA.
(TIF)

S21 Fig Rank of 38 tissues in the GTEx data in terms of their relevance to each of the eight GWAS traits obtained using CoCoNet. Here, based on the analysis in the main text, we used the test statistics of the per-SNP heritability as outcome in the model. For neurological diseases (**A-D**) and autoimmune diseases (**E-H**), we calculated the composite likelihood for each tissue, subtracted the minimum likelihood across all tissues (x-axis), and ranked traits based on these values from top to bottom in each panel (y-axis). The brain tissues are colored in blue; the colon related tissues are colored in yellow; and the rest of the tissues are colored in green. Consistent with the results when using per-SNP heritability as outcome, brain tissues tend to rank high for the four neurological diseases (top four panels) while colon related tissues tend to rank high for autoimmune diseases (bottom four panels).
(TIF)

S22 Fig Reproducibility score for ranked tissues in simulations. The reproducibility score (y-axis) is computed for each ordered tissue rank (x-axis) in each simulation replicate and averaged across replications. Results are shown for simulation scenarios I (**A**), II (**B**), and III (**C**). The reproducibility score is high for top ranked tissues while gradually reduces to zero with increasing rank order. In simulation scenario I, II, and III, we set the signal strength to be 0.02, and in scenario II and III, we set the perturbation strength to be 1%.
(TIF)

S23 Fig Reproducibility score for 38 tissues in the real data. Results are shown for 4 neurological traits (**A-D**) and 4 autoimmune diseases (**E-H**). For each trait, the reproducibility score (y-axis) is computed for ordered rank (x-axis). For most traits, the reproducibility score is reasonably high for top ranked tissues.
(TIF)

S24 Fig Compare rank of 38 tissues in the original data versus that in the subsampled data. Results are shown for 4 neurological traits (**A-D**) and 4 autoimmune diseases (**E-H**). For each GWAS trait, we randomly removed 10% of connected gene pairs in the tissue specific network and constructed 10 submatrices. The rank of the tissues in the original data (x-axis) is then compared with their rank in the 10 subsampled data (y-axis). For most traits, the top ranked tissues have relatively stable rank in the subsampled data while the lowly ranked tissues have highly variable ranks in the subsampled data.
(TIF)

S25 Fig Rank of 38 tissues in the GTEx data in terms of their relevance to additional GWAS traits obtained using CoCoNet. We collected additional four GWAS traits, including body mass index (BMI), height, waist-to-hip ratio (WHR) from the GIANT consortium, and Coronary Artery Disease (CAD) from the CARDIoGRAM consortium. For each GWAS trait, we calculated the composite likelihood for each tissue, subtracted the minimum likelihood across all tissues (x-axis), and ranked traits based on these values from top to bottom in each panel (y-axis). In BMI, brain tissues are colored in blue, and the other tissues are colored in yellow (**A**); in height, muscle tissue is colored in blue, and the other tissues are colored in yellow (**B**); in WHR, the adipose tissues are colored in blue, the muscle tissue is colored in green, the colon tissues are colored in yellow, and the other tissues are colored in red (**C**); in CAD, the artery tissues are colored in blue, and the other tissues are colored in yellow (**D**). The results are largely consistent with previous literatures.
(TIF)

S26 Fig Compare rank of 38 tissues in the original data versus that in the subsampled data. Results are shown For each of the additional four GWAS trait including body mass index (BMI), height, waist-to-hip ratio (WHR) from the GIANT consortium, and Coronary Artery Disease (CAD) from the CARDIoGRAM consortium in (**A-D**). For each GWAS trait, we randomly removed 10% of connected gene pairs in the tissue specific network and constructed 10 submatrices. The rank of the tissues in the original data (x-axis) is then compared with their rank in the 10 subsampled data (y-axis). For most traits, the top ranked tissues have relatively stable rank in the subsampled data while the lowly ranked tissues have highly variable ranks in the subsampled data. The Reproducibility score for 38 tissues in the real data are shown in (**E-H**). For each trait, the reproducibility score (y-axis) is computed for ordered rank (x-axis). For most traits, the reproducibility score is reasonably high for top ranked tissues.
(TIF)

S1 Table Computation time for CoCoNet in the two real data applications. Computing time is based on analysis of each trait-tissue pair using a single thread on a Xeon CPU E5-2620 v2 @ 2.10GHz processor.
(XLSX)

S2 Table Information for the summary statistics of eight GWAS traits. (XLSX)

S3 Table PubMed search keywords for eight GWAS traits. (XLSX)

S4 Table PubMed search keywords for 38 tissues. (XLSX)

S5 Table PubMed search keywords for 10 cell types. (XLSX)

S6 Table Reproducibility score of the top ranking tissues in 8 GWAS traits. (XLSX)

S1 Text Supplementary text for the methods. (DOCX)
