# Supplementary figures and images for: Leveraging gene co-expression patterns to infer trait-relevant tissues in genome-wide association studies
Source: PLoS Genet. 2020 Apr 20;16(4):e1008734. doi: 10.1371/journal.pgen.1008734 (PMC7192514; doi:10.1371/journal.pgen.1008734)

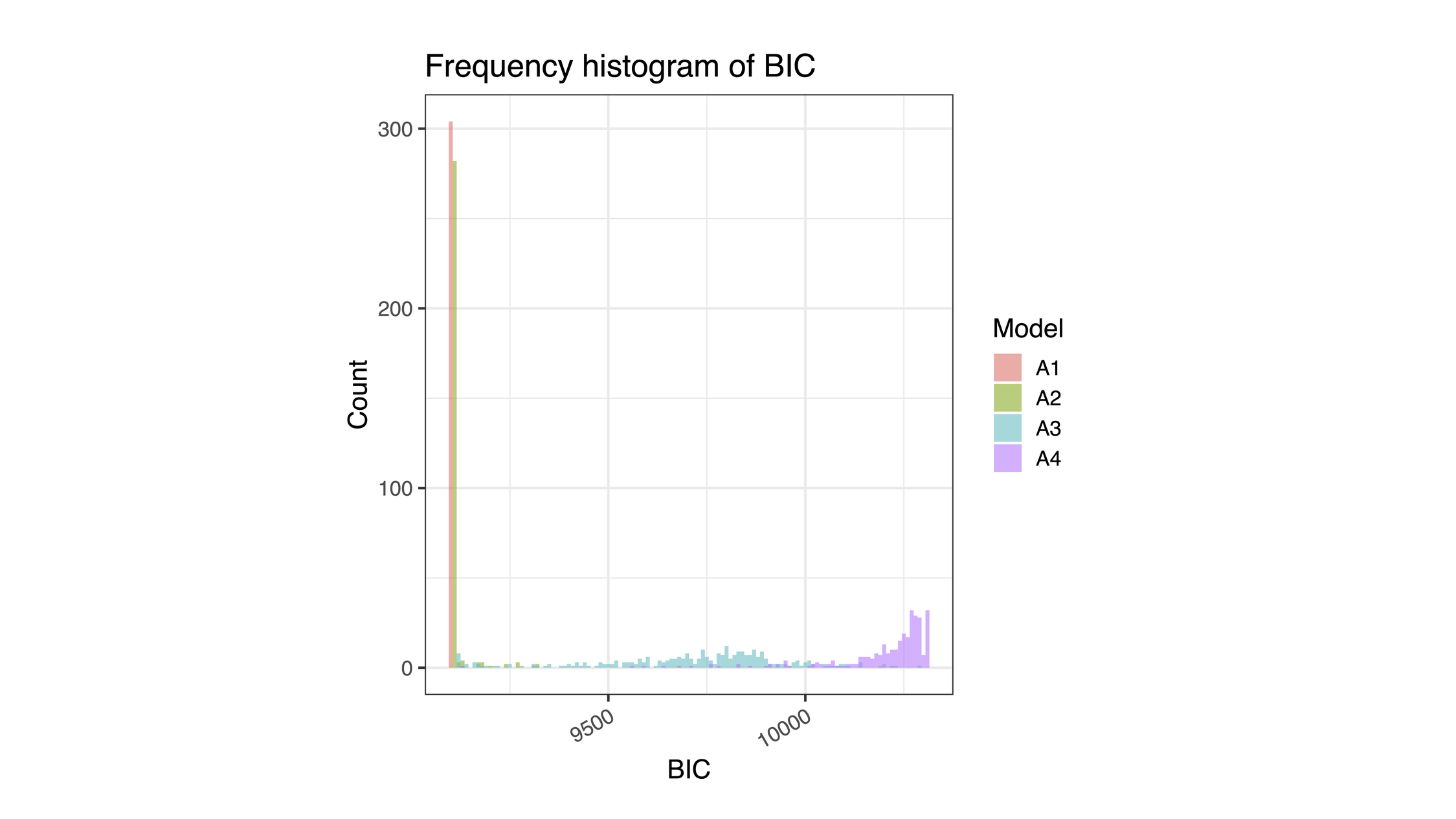

Supplement: S1 Fig — Here, K is the number of matrices included in the covariance function. In the real data application, we analyzed all pairs of 38 GTEx tissues and 8 GWAS traits. For each of the 304 trait-tissue pairs, we fit four different CoCoNet models with K ranging from 1 to 4. The above histogram shows the Bayesian information criterion (BIC) values (x-axis) across all these CoCoNet models. Models with different K are colored differently: A1 represents a model with 1st power of the adjacency matrix (red); A2 represents a model with both 1st and 2nd power of the adjacency matrix (green); A3 represents a model with up to the 3rd power of the adjacency matrix (blue); A4 represents a model with up to the 4th power of the adjacency matrix (purple). The results suggest that a model with a low value of K (1 or 2) often comes with a lower BIC and is thus often preferred than a model with a high K. (TIF) [file pgen.1008734.s001.tif]

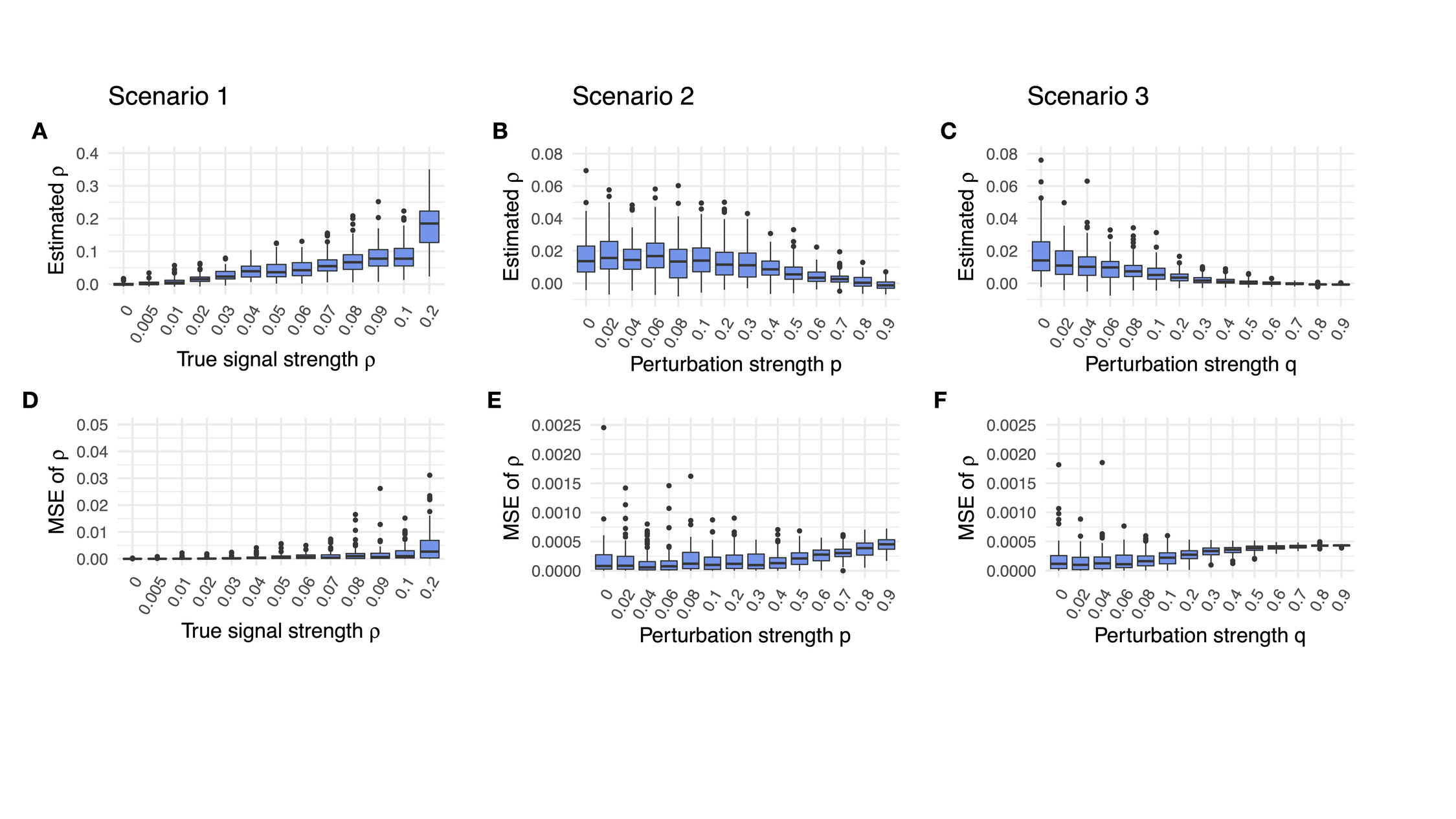

Supplement: S2 Fig — (A): Boxplot shows the ρ estimates across 100 simulation replicates for each true ρ (x-axis) in the simulation scenario I. (B): Boxplot shows the ρ estimates across 100 simulation replicates for each parameter p (x-axis) in the simulation scenario II. Here, true ρ = 0.02. Increasing parameter p adds increasingly large noise to the tissue-specific adjacency matrices, thus leading to downward biased estimation of ρ. (C): Boxplot shows the ρ estimates across 100 simulation replicates for each parameter q (x-axis) in the simulation scenario III. Here, true ρ = 0.02. Increasing parameter q adds increasingly more noise to the tissue-specific adjacency matrices, thus leading to downward biased estimation of ρ. (D-F): mean squared error (MSE; y-axis) measures the accuracy of ρ estimates across the three simulation scenarios. (TIF) [file pgen.1008734.s002.tif]

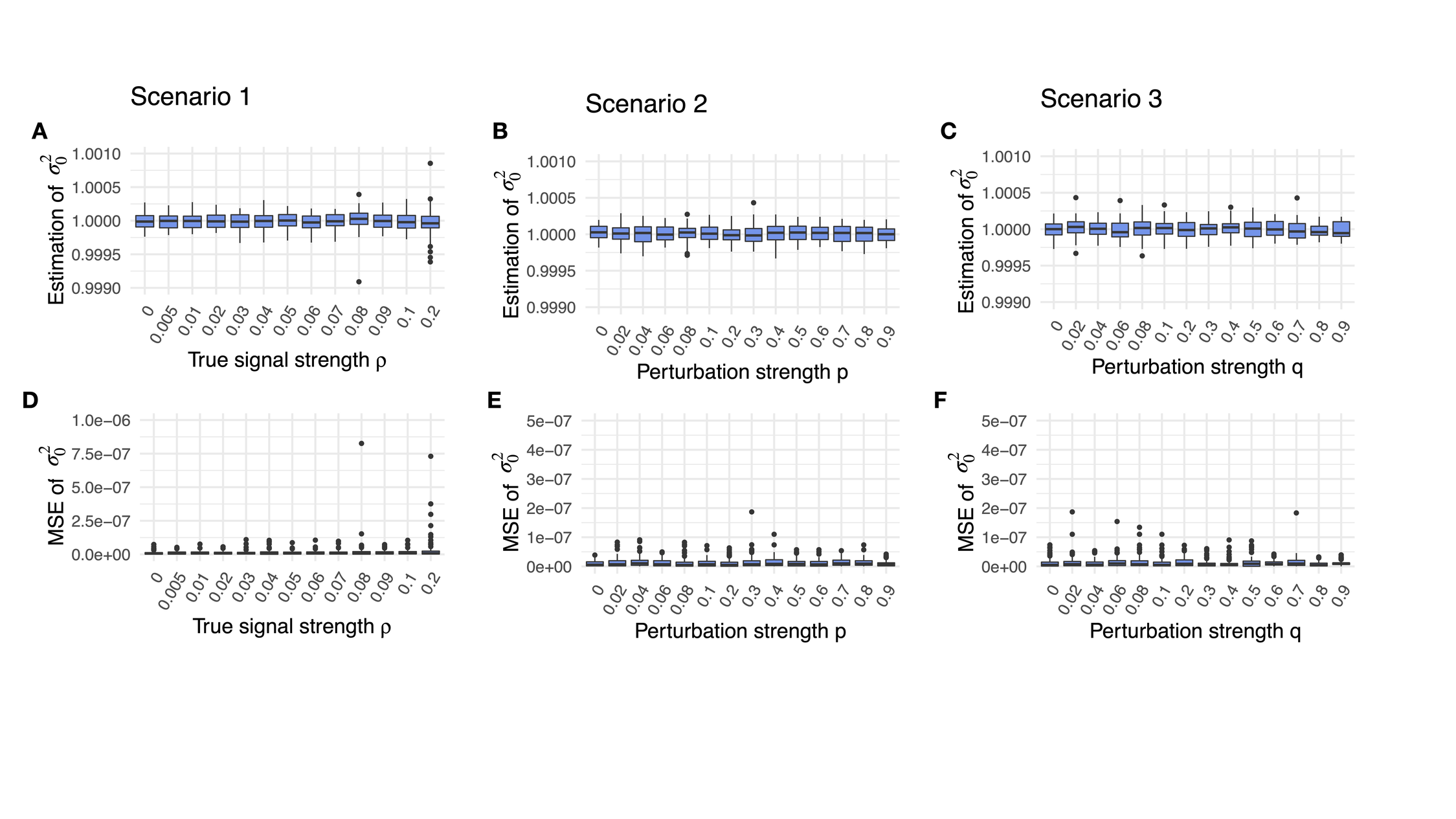

Supplement: S3 Fig — (A): Boxplot shows the σ02 estimates across 100 simulation replicates for each signal strength parameter ρ (x-axis) in the simulation scenario I. (B): Boxplot shows the σ02 estimates across 100 simulation replicates for each parameter p (x-axis) in the simulation scenario II. Here, true σ02=0.98. Increasing parameter p adds increasingly large noise to the tissue-specific adjacency matrices, but does not appear to strongly influence the estimation of σ02. (C): Boxplot shows the σ02 estimates across 100 simulation replicates for each parameter q (x-axis) in the simulation scenario III. Here, true σ02=0.98. Increasing parameter q adds increasingly large noise to the tissue-specific adjacency matrices, but does not appear to strongly influence the estimation of σ02. (D-F): mean squared error (MSE; y-axis) measures the accuracy of σ02 estimates across the three simulation scenarios. (TIF) [file pgen.1008734.s003.tif]

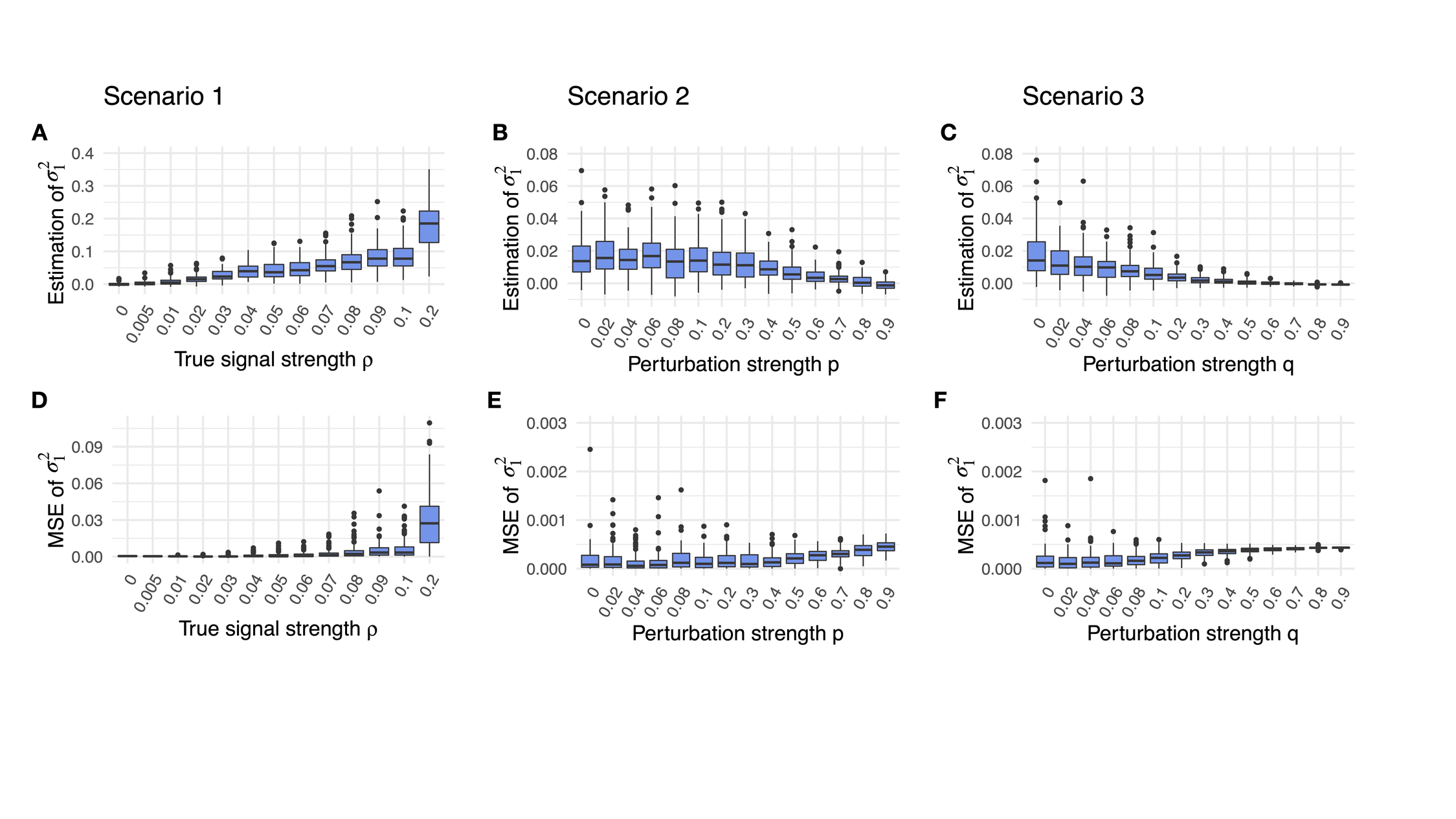

Supplement: S4 Fig — (A): Boxplot shows the σ12 estimates across 100 simulation replicates for each signal strength parameter ρ (x-axis) in the simulation scenario I. (B): Boxplot shows the σ12 estimates across 100 simulation replicates for each parameter p (x-axis) in the simulation scenario II. Here, true σ12=0.02. Increasing parameter p adds increasingly large noise to the tissue-specific adjacency matrices, but does not appear to strongly influence the estimation of σ12. (C): Boxplot shows the σ12 estimates across 100 simulation replicates for each parameter q (x-axis) in the simulation scenario III. Here, true σ12=0.02. Increasing parameter q adds increasingly large noise to the tissue-specific adjacency matrices, but does not appear to strongly influence the estimation of σ12. (D-F): mean squared error (MSE; y-axis) measures the accuracy of σ12 estimates across the three simulation scenarios. (TIF) [file pgen.1008734.s004.tif]

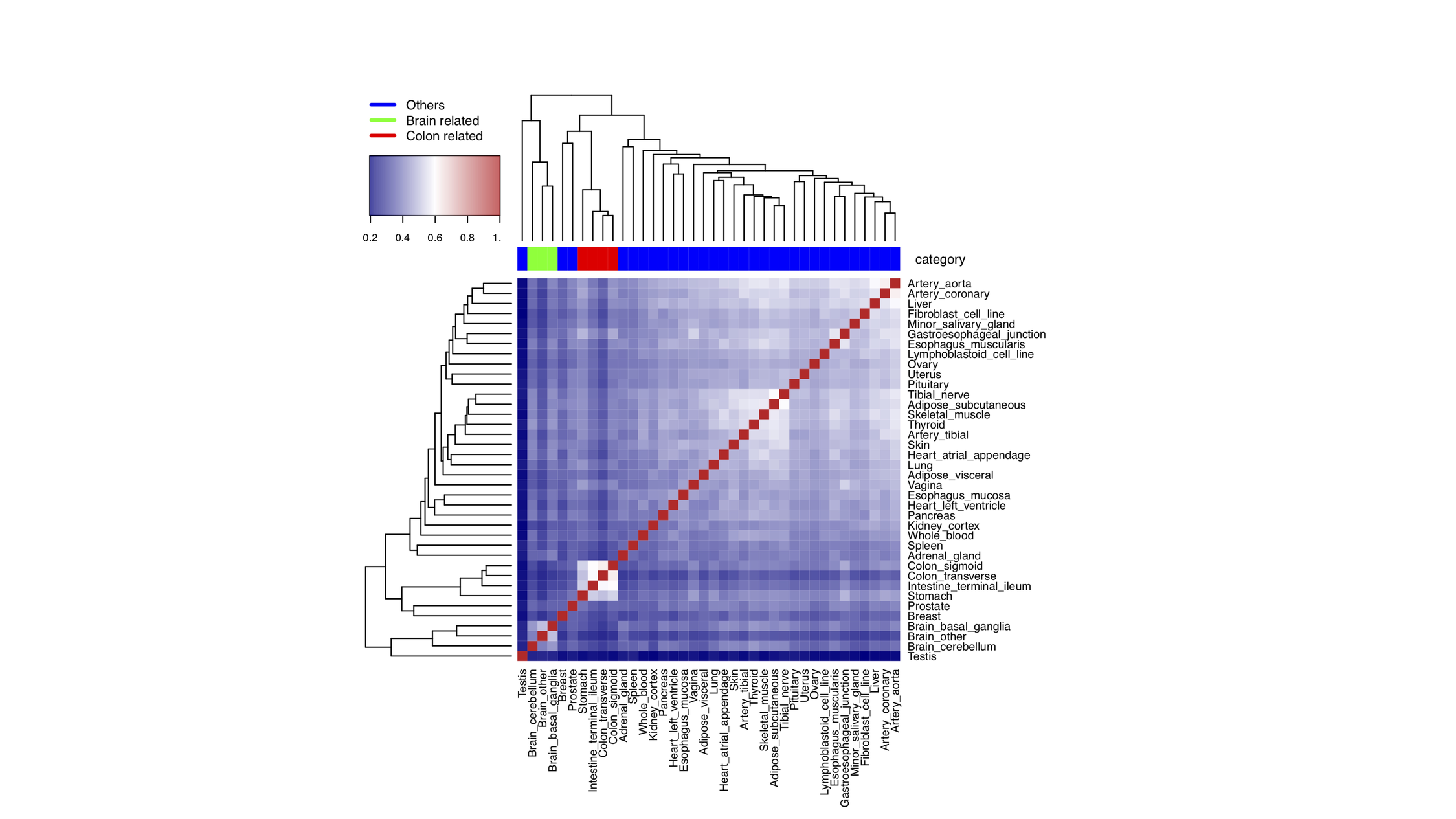

Supplement: S5 Fig — Jaccard index is computed between pairs of matrices to measure the similarity among adjacency matrices across tissues. Adjacency matrices on similar tissues tend to cluster together based on hierarchical clustering. For example, the adjacency matrices for the three brain tissues (green), such as basal ganglia, cerebellum, and brain other, are all clustered together. Similarly, intestinal tissues (red), such as stomach, colon-transverse, intestine terminal ileum, and colon sigmoid, are all clustered together. (TIF) [file pgen.1008734.s005.tif]

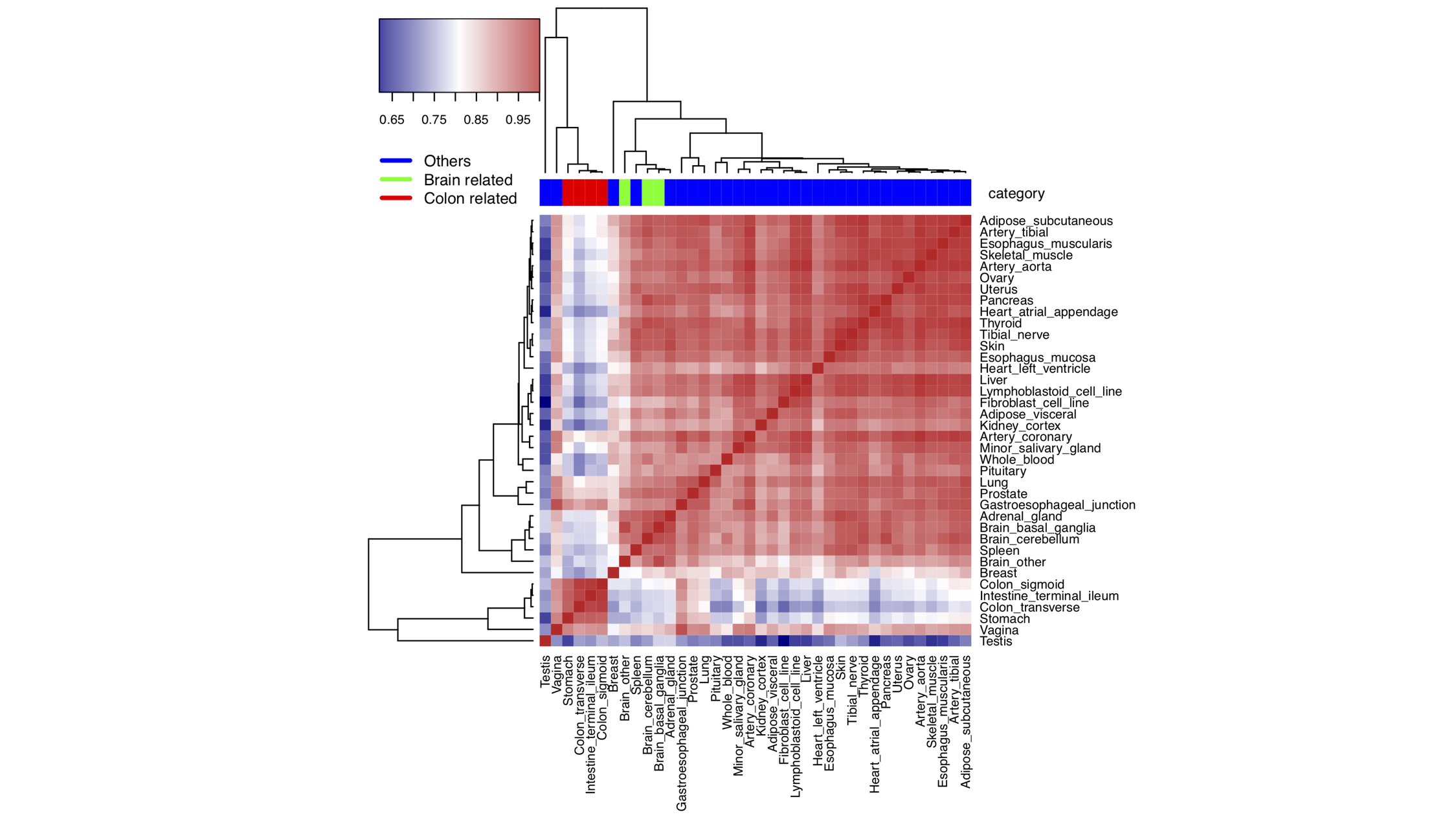

Supplement: S6 Fig — In each tissue, we calculated for each gene a node connectivity value, which measures the number of genes it is directly connected to a gene with a high node connectivity value is often referred to as a hub gene. Node connectivity values are similar between similar tissues as measured by Pearson’s correlation; thus similar tissues tend to cluster together based on Pearson’s correlation. (TIF) [file pgen.1008734.s006.tif]

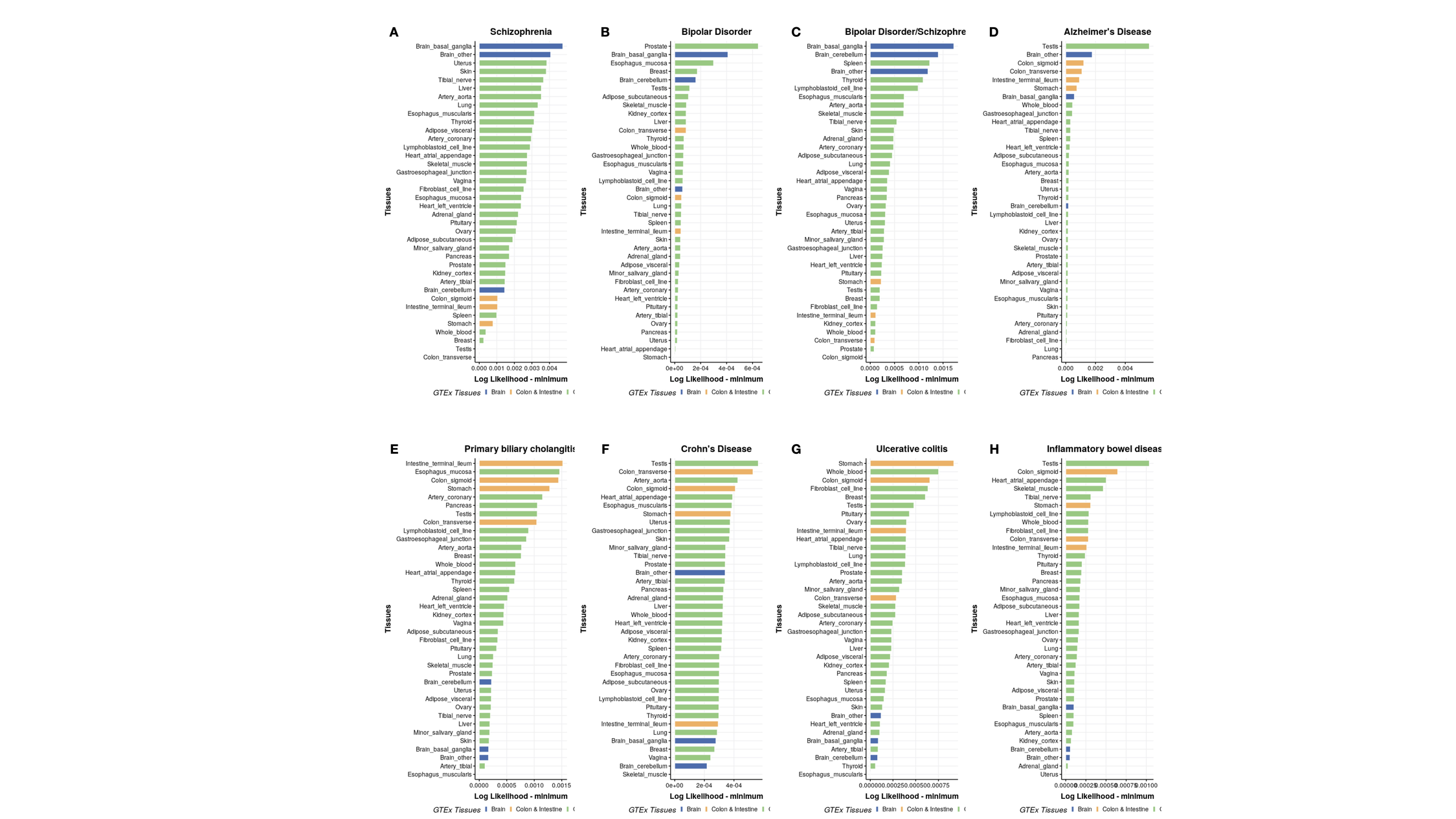

Supplement: S7 Fig — For each GWAS trait, we calculated the composite likelihood for each tissue, subtracted the minimum likelihood across all tissues (x-axis), and ranked traits based on these values from top to bottom in each panel (y-axis). The brain tissues are colored in blue; the colon related tissues are colored in yellow; and the rest of the tissues are colored in green. Brain tissues tend to rank high for the four neurological diseases (A-D) while colon related tissues tend to rank high for autoimmune diseases (E-H). (TIF) [file pgen.1008734.s007.tif]

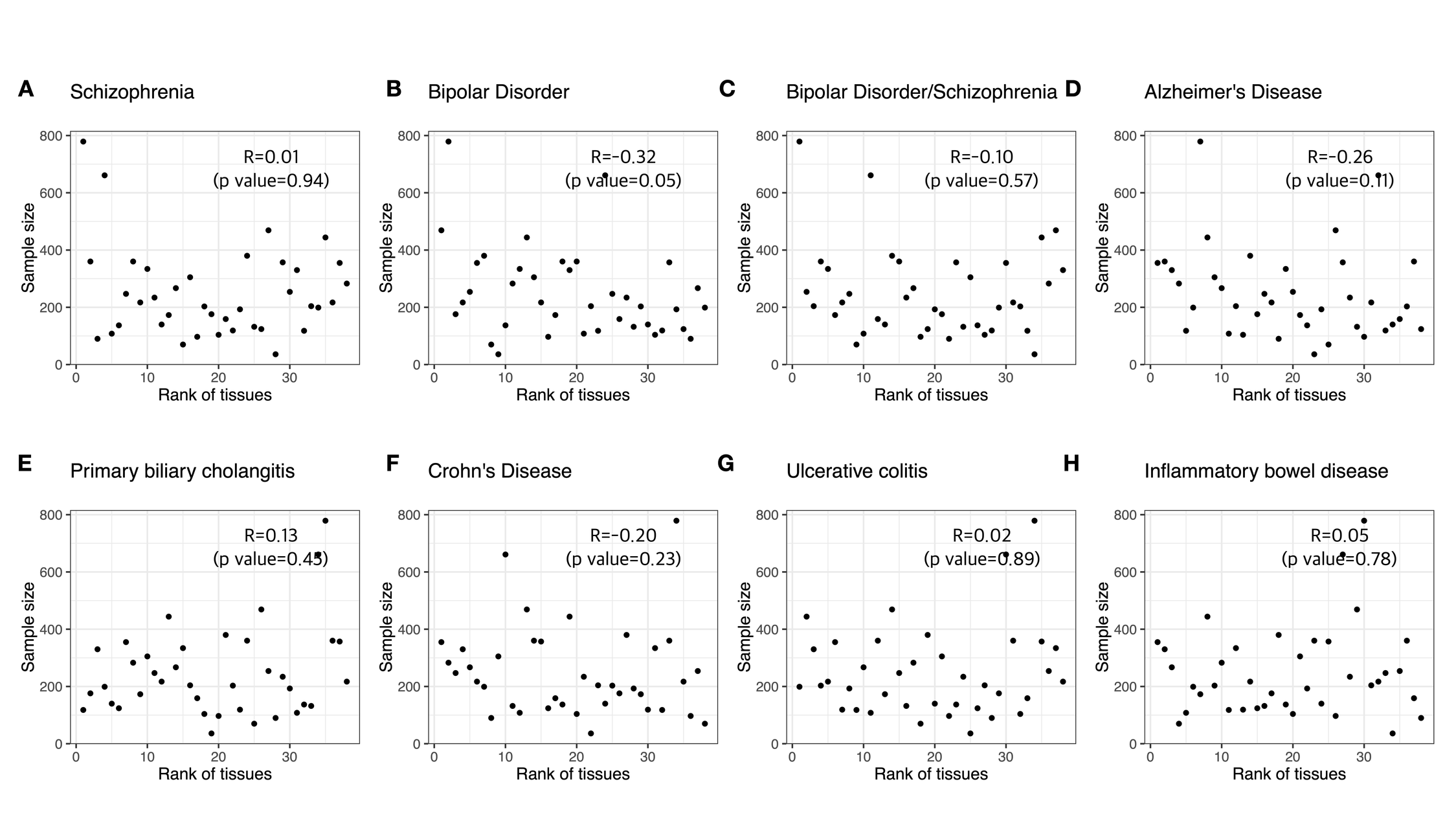

Supplement: S8 Fig — The sample size of each tissue (y-axis) is plotted against the rank of each tissue by CoCoNet (x-axis) across eight GWAS traits (eight panels). Spearman’s rank correlation (R) between the tissue rank and the sample size, together with the corresponding p-value, are also displayed on the panels for neurological diseases (A-D) and autoimmune diseases (E-H). The tissue rank obtained by CoCoNet is not correlated with tissue sample size for all traits. (TIF) [file pgen.1008734.s008.tif]

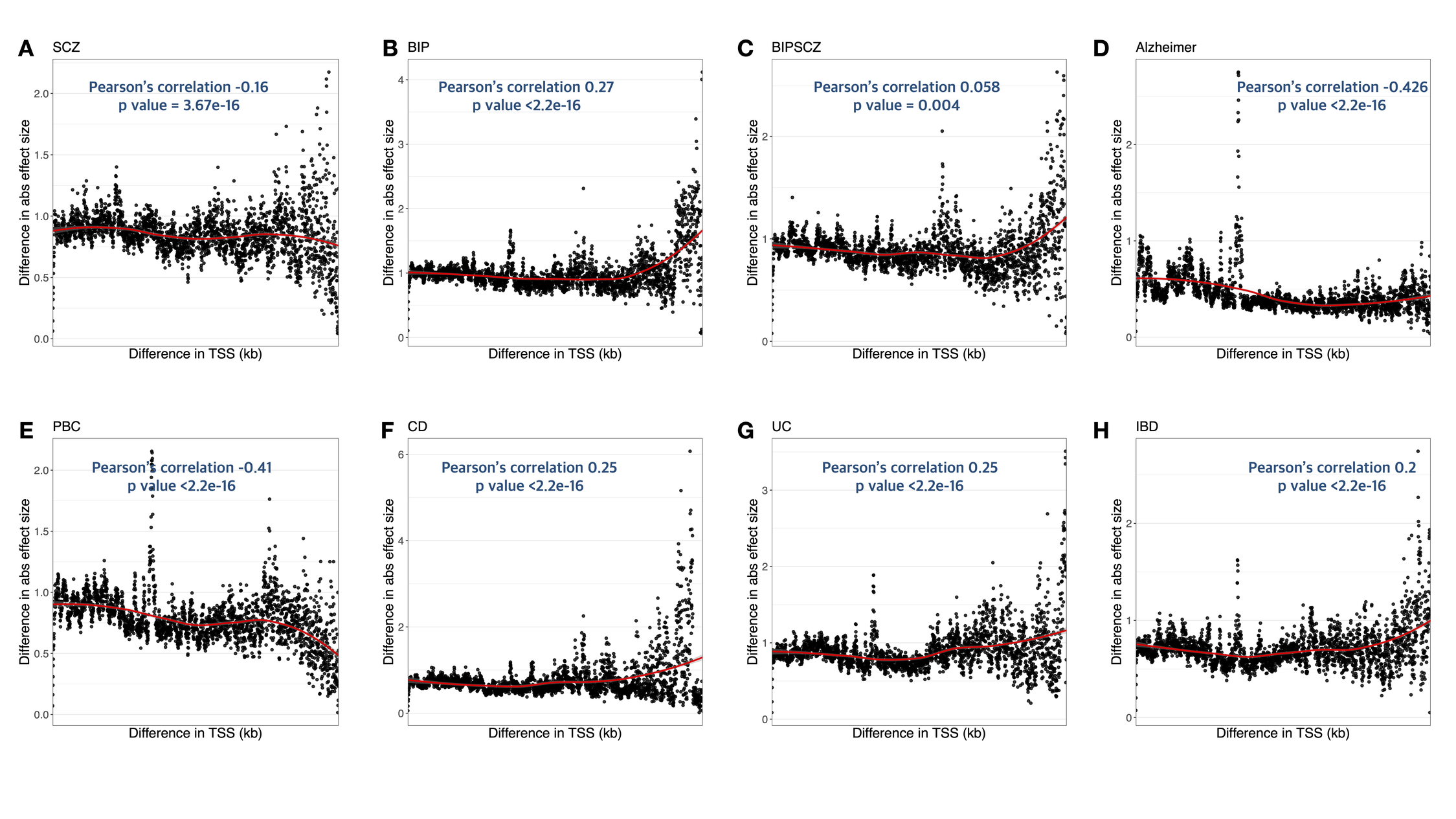

Supplement: S9 Fig — Difference in effect measurement between two genes in a pair is measured by the absolute difference in per-SNP gene-level heritability (y-axis). Distance between two genes in a pair is measured by their TSS locations (x-axis). The Pearson’s correlation between the gene distance differences and the absolute gene effect measurements difference is calculated for neurological diseases (A-D) and autoimmune diseases (E-H). (TIF) [file pgen.1008734.s009.tif]

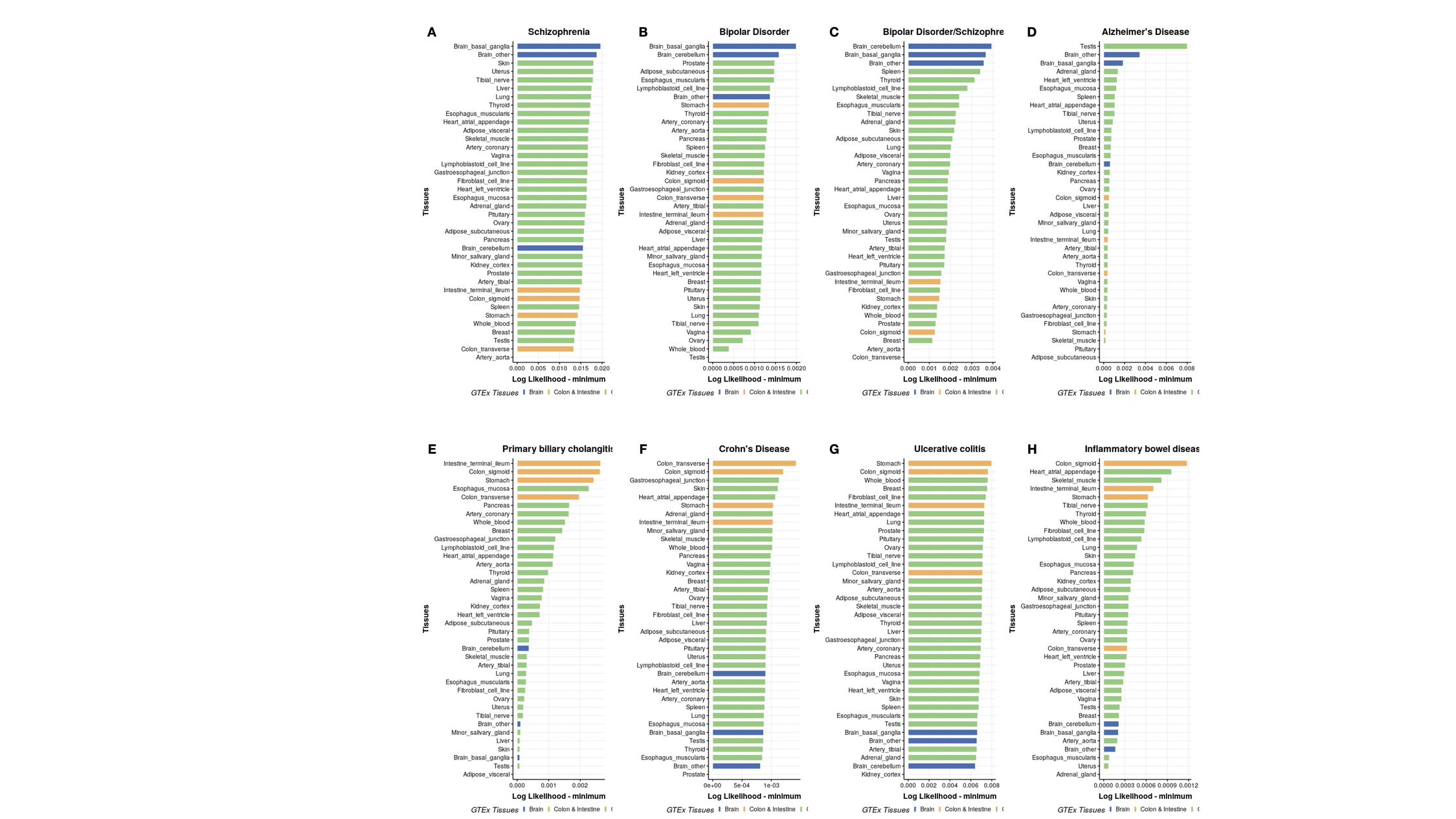

Supplement: S10 Fig — Here, based on the analysis in the main text, we use the distance matrix for genes as covariance. The distances between genes are measured by differences in transcript starting site (TSS) locations, then scaled by the maximum distance between genes. The distances between genes on different chromosomes are defined as 1 in the scaled distance matrix. For neurological diseases (A-D) and autoimmune diseases (E-H), we calculated the composite likelihood for each tissue, subtracted the minimum likelihood across all tissues (x-axis), and ranked traits based on these values from top to bottom in each panel (y-axis). The brain tissues are colored in blue; the intestinal related tissues are colored in yellow; and the rest of the tissues are colored in green. The results are highly consistent with the results in main text. (TIF) [file pgen.1008734.s010.tif]

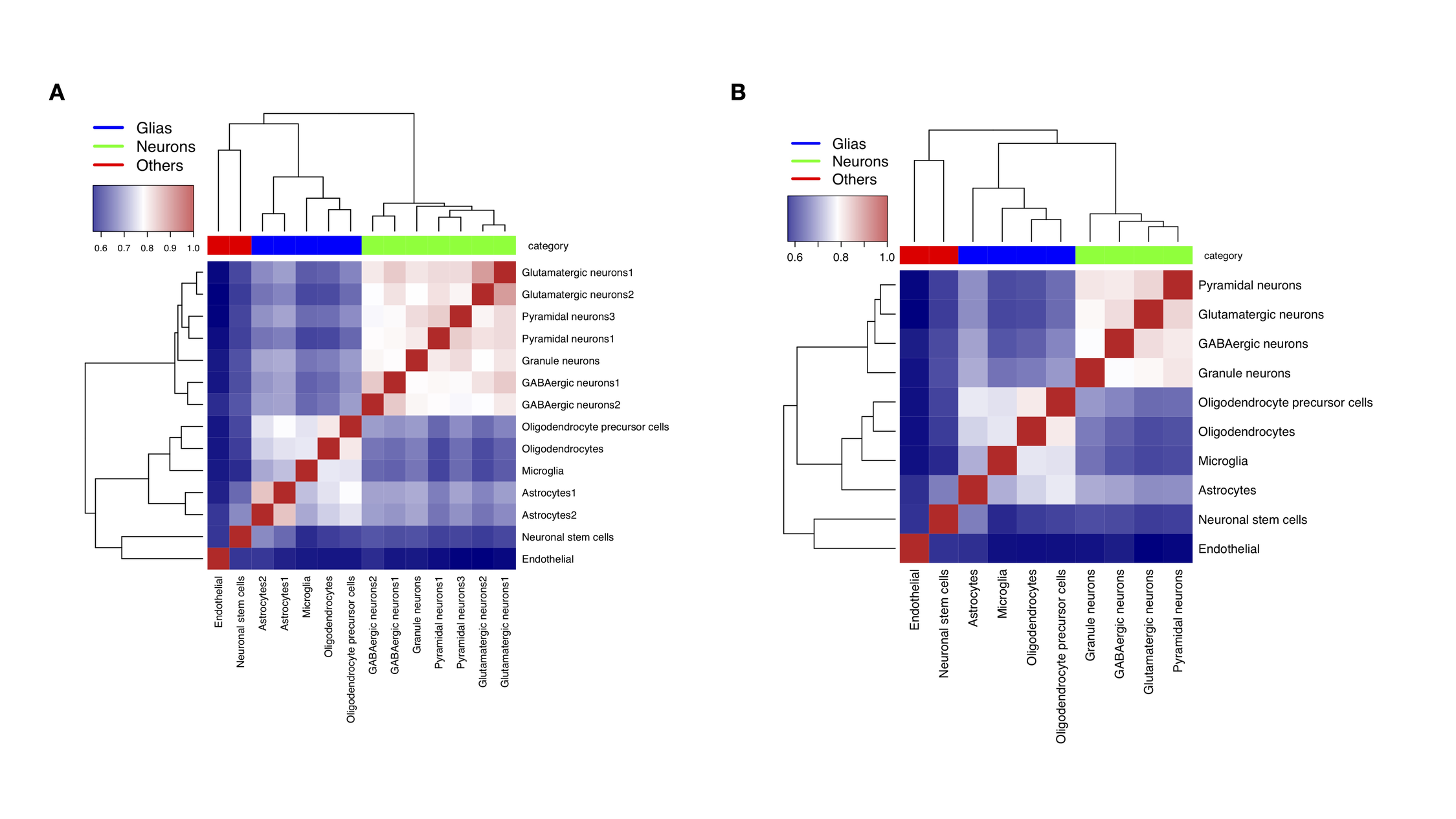

Supplement: S11 Fig — Jaccard index between adjacency matrices constructed either from cell types in two donors separately (A) or from cell types merged from two donors (B) is measured by Jaccard index. Adjacency matrices on similar tissues tend to cluster together based on hierarchical clustering. For example, the same cell type from different donors tend to cluster together (A) and different glia cell types tend to cluster together (B). The Jaccard index between two identical matrices is 1, as shown on the diagonal with red color in the heatmap. (TIF) [file pgen.1008734.s011.tif]

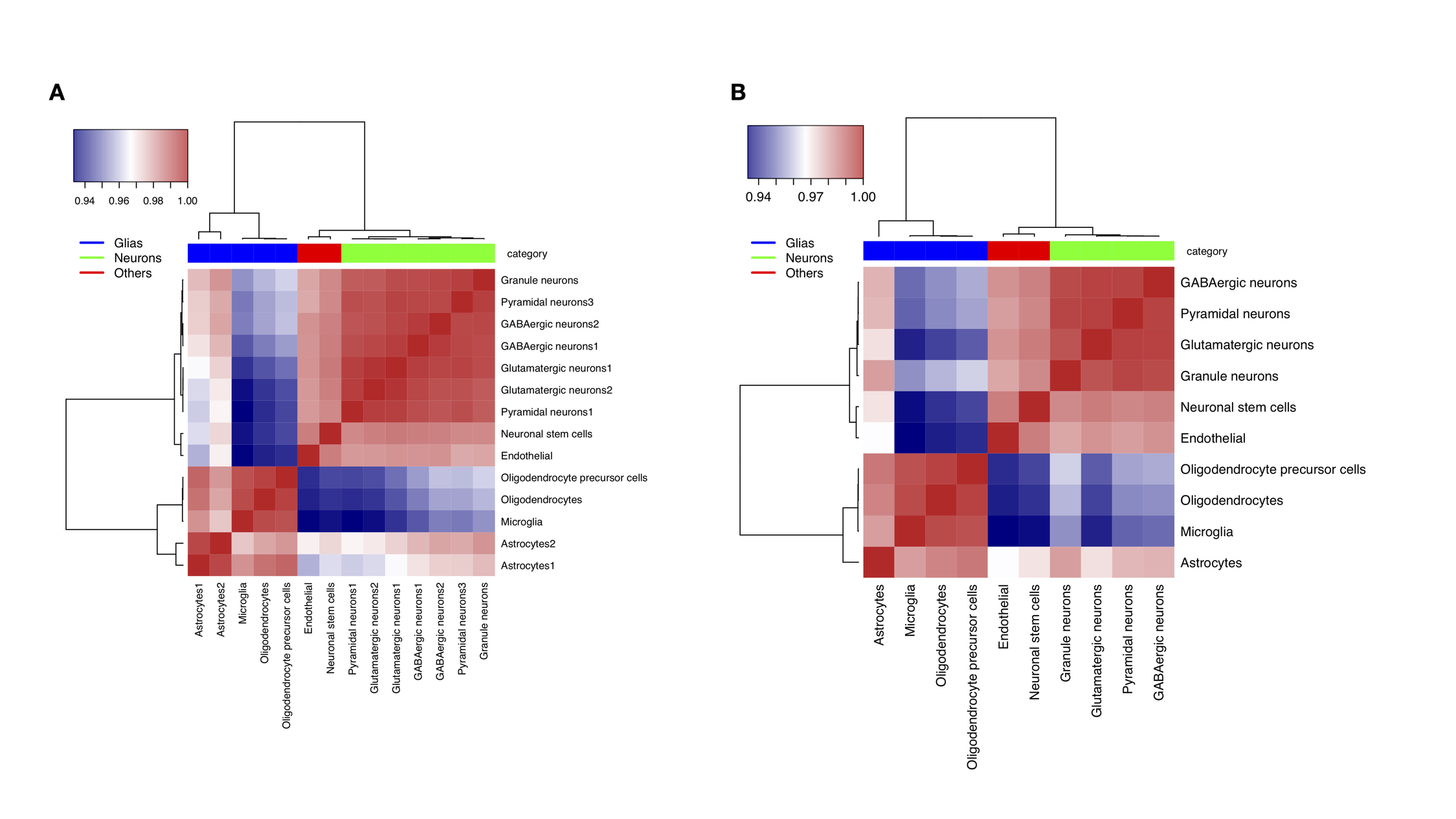

Supplement: S12 Fig — We calculated for each gene a node connectivity value, which measures the number of genes it is directly connected to a gene with a high node connectivity value is often referred to as a hub gene. Similarity between cell types constructed either from two donors separately (A) or from cell types merged from two donors (B) is measured by Pearson’s correlation. The Pearson’s correlation between two identical matrices is 1, as shown on the diagonal with red color in the heatmap. (TIF) [file pgen.1008734.s012.tif]

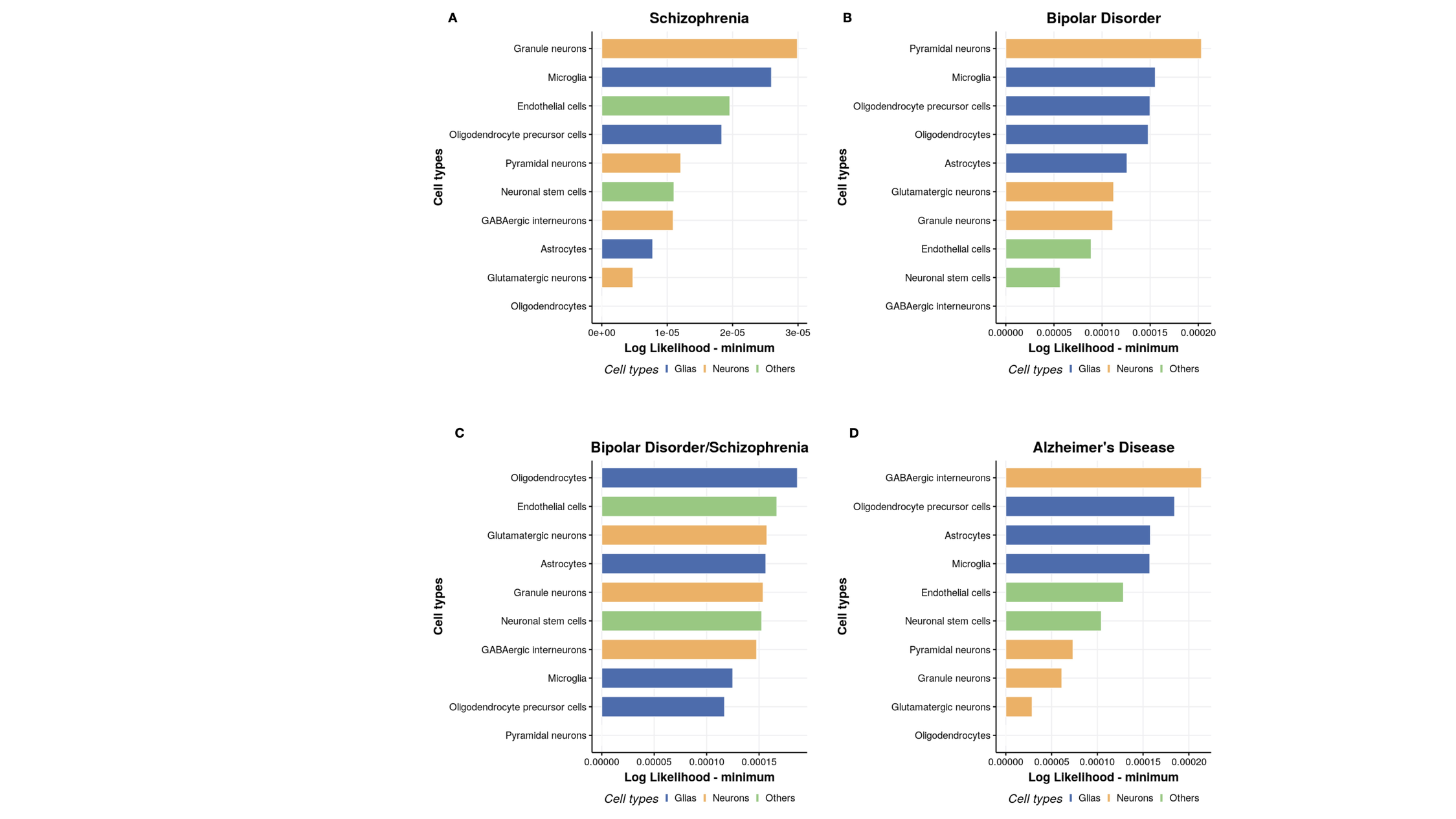

Supplement: S13 Fig — The number of cells in each tissue (y-axis) is plotted against the rank of each cell type by CoCoNet (x-axis) across neurological diseases (A-D). Spearman’s rank correlation (R) between the cell type rank and the number of cells, together with the corresponding p-value, are also displayed on the panels. The cell type rank obtained by CoCoNet is not correlated with the number of cells in the cell type for all traits. (TIF) [file pgen.1008734.s013.tif]

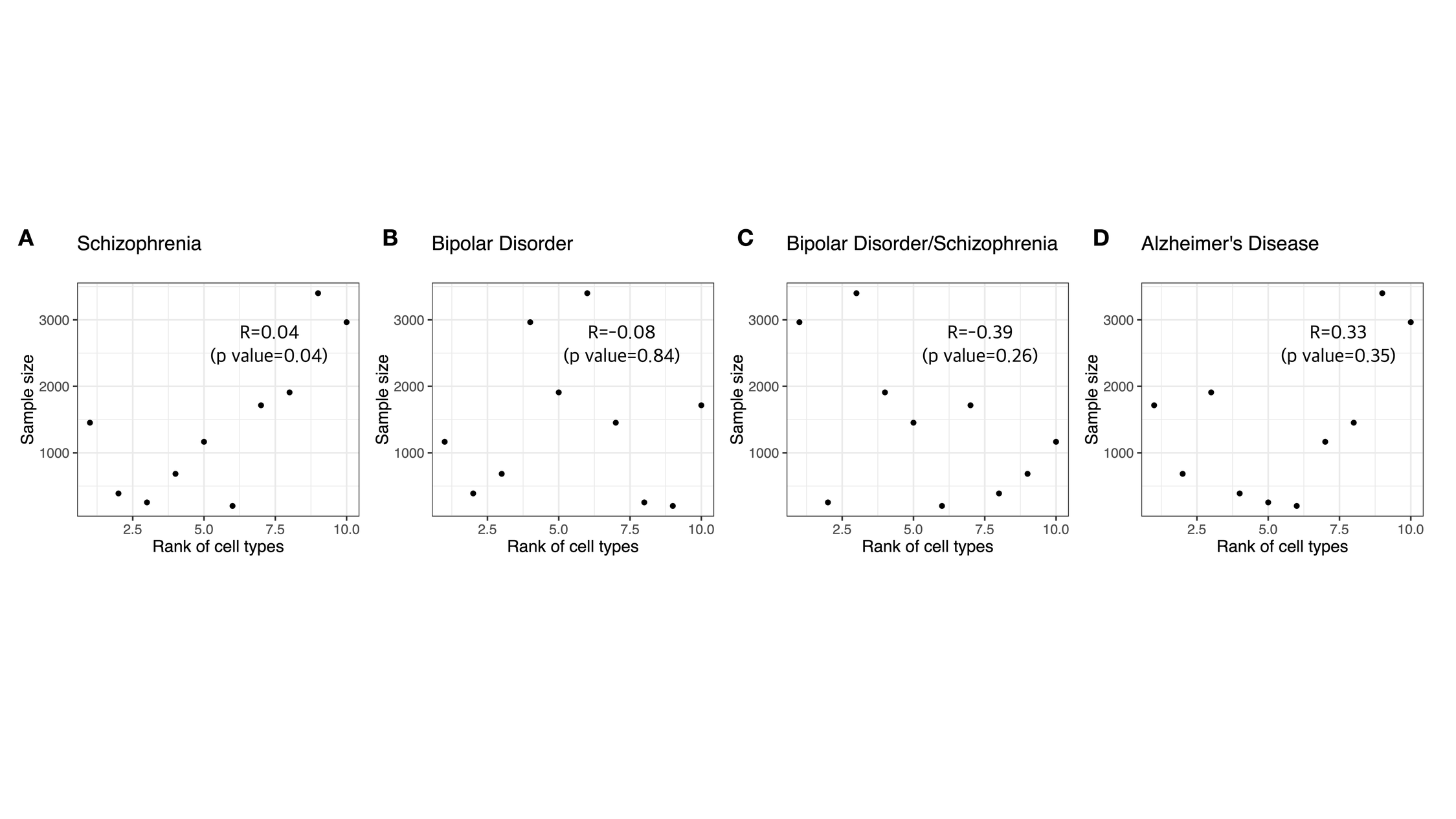

Supplement: S14 Fig — For each of the four GWAS trait (A-D), we calculated the composite likelihood for each cell type, subtracted the minimum likelihood across all cell types (x-axis), and ranked cell types based on these values from top to bottom in each panel (y-axis). (TIF) [file pgen.1008734.s014.tif]

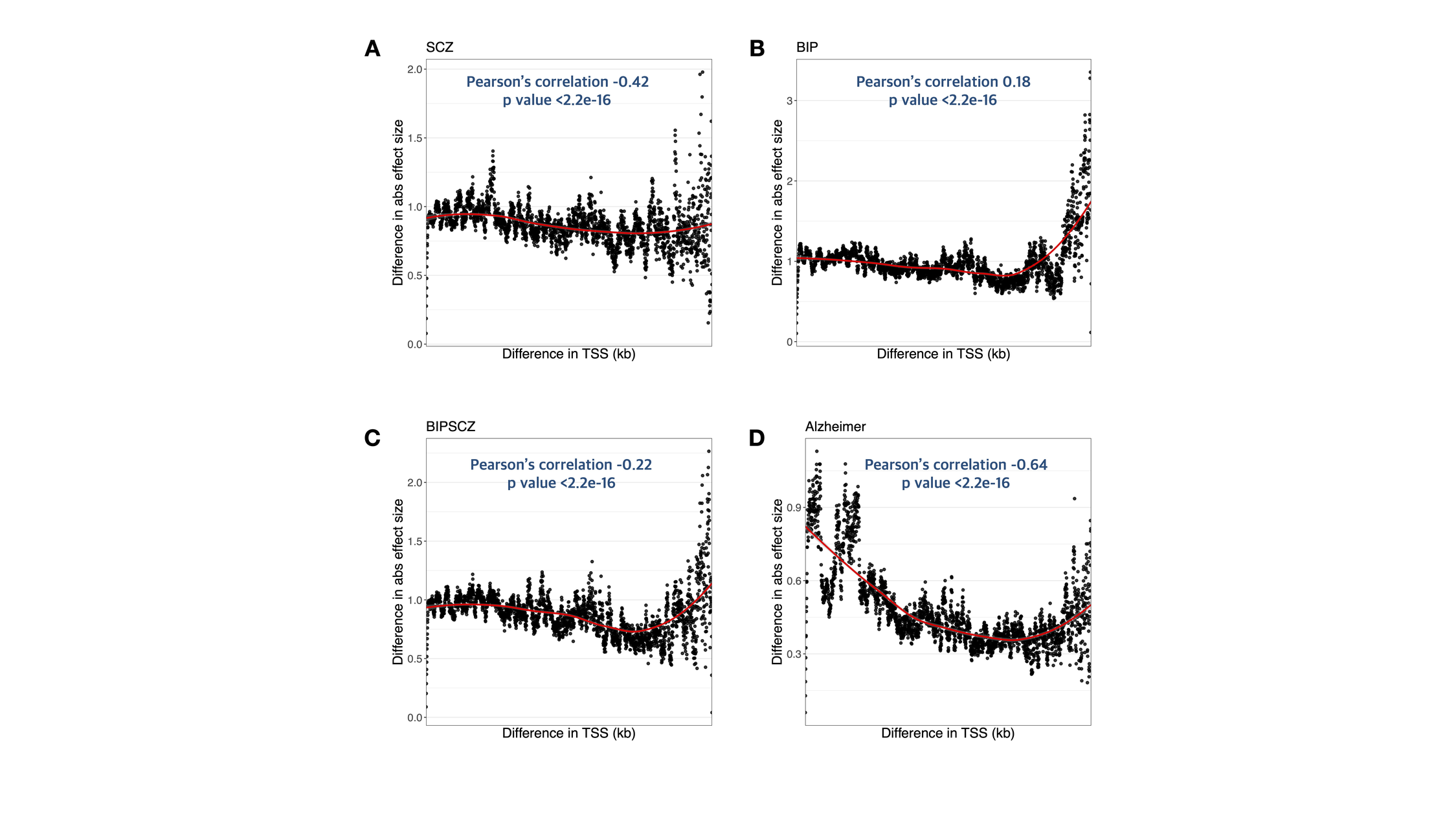

Supplement: S15 Fig — Difference in effect measurement between two genes in a pair is measured by the absolute difference in per-SNP gene-level heritability (y-axis). Distance between two genes in a pair is measured by their TSS locations (x-axis). The Pearson’s correlation between the gene distance differences and the absolute gene effect measurements difference is calculated for each GWAS trait (A-D). (TIF) [file pgen.1008734.s015.tif]

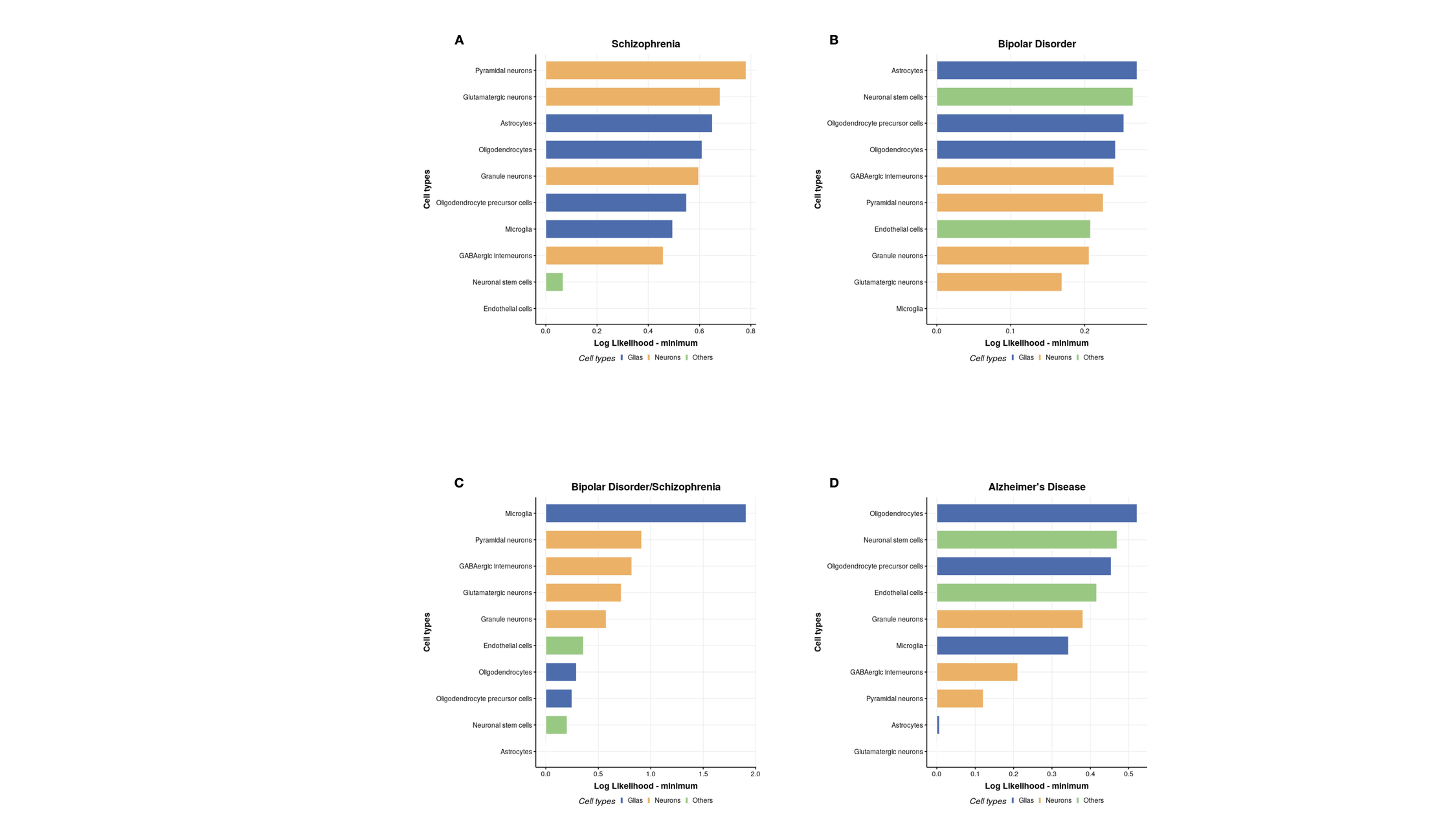

Supplement: S16 Fig — Here, we use the distance matrix for genes as covariance. The distances between genes are measured by differences in transcript starting site (TSS) locations, then scaled by the maximum distance between genes. The distances between genes on different chromosomes are defined as 1 in the scaled distance matrix. For each of the four GWAS trait (A-D), we calculated the composite likelihood for each cell type, subtracted the minimum likelihood across all cell types (x-axis), and ranked cell types based on these values from top to bottom in each panel (y-axis). (TIF) [file pgen.1008734.s016.tif]

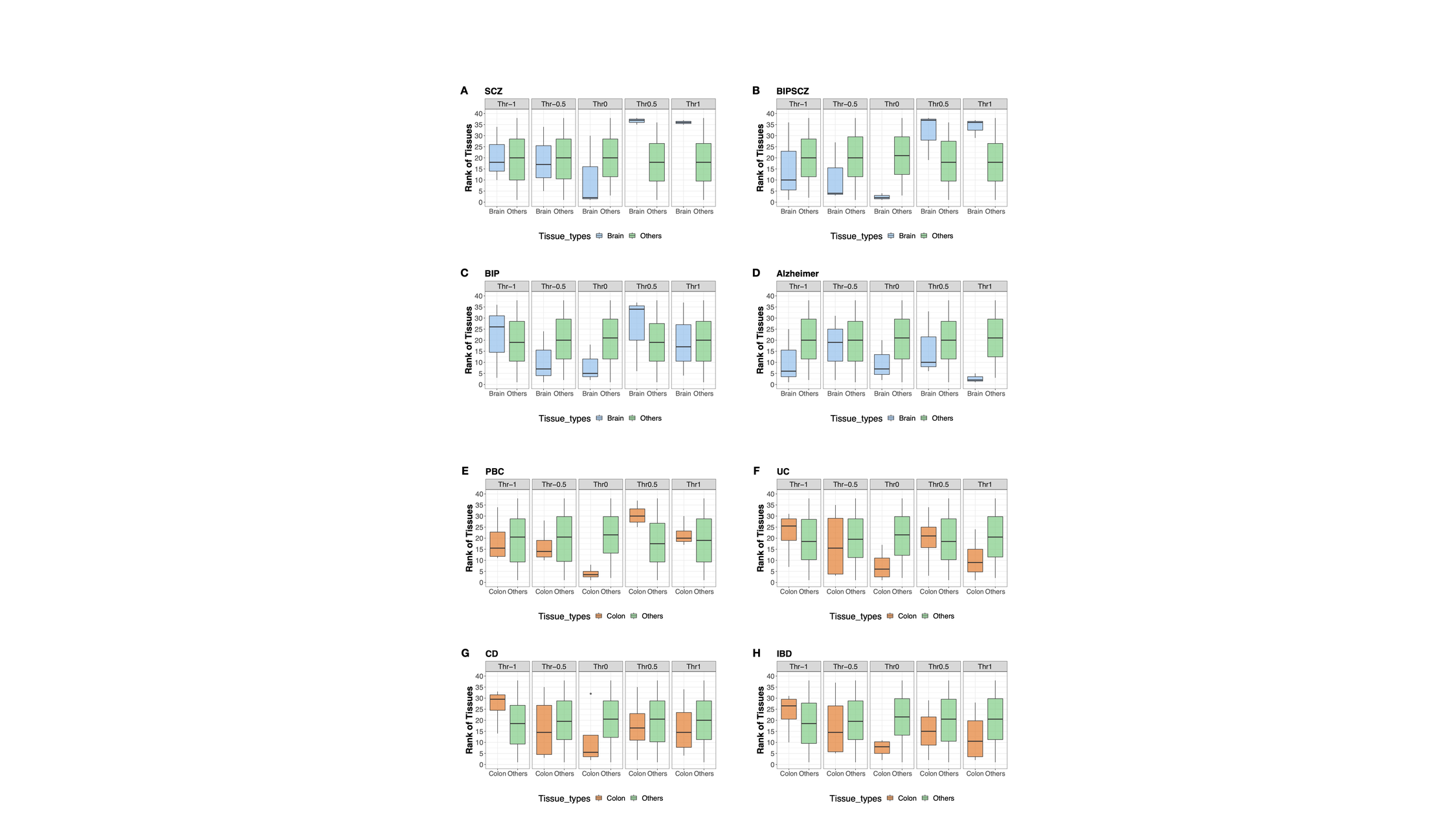

Supplement: S17 Fig — In our main analysis, we performed a hard thresholding procedure to convert the continuous edge values to binary values. In particular, edges that had a positive edge value and that were specific to at least one tissue type were converted to one; otherwise, they were converted to zero. Here, we varied such hard threshold and found the trait-tissue relevant results are relatively robust regardless of what hard thresholds we used. (A-D): For each of the four neurological diseases, we plotted the rank (y-axis) of three brain tissues (colored blue; including brain cerebellum, brain basal ganglia, and brain other) and the rank of the remaining 35 tissues (colored green) in separate boxplots. (E-H): For each of the four autoimmune diseases, we plotted the rank (y-axis) of four intestinal tissues (colored orange; including colon sigmoid, colon transverse, and intestine terminal ileum, stomach) and the rank of the remaining 34 tissues (colored green) in separate boxplots. (TIF) [file pgen.1008734.s017.tif]

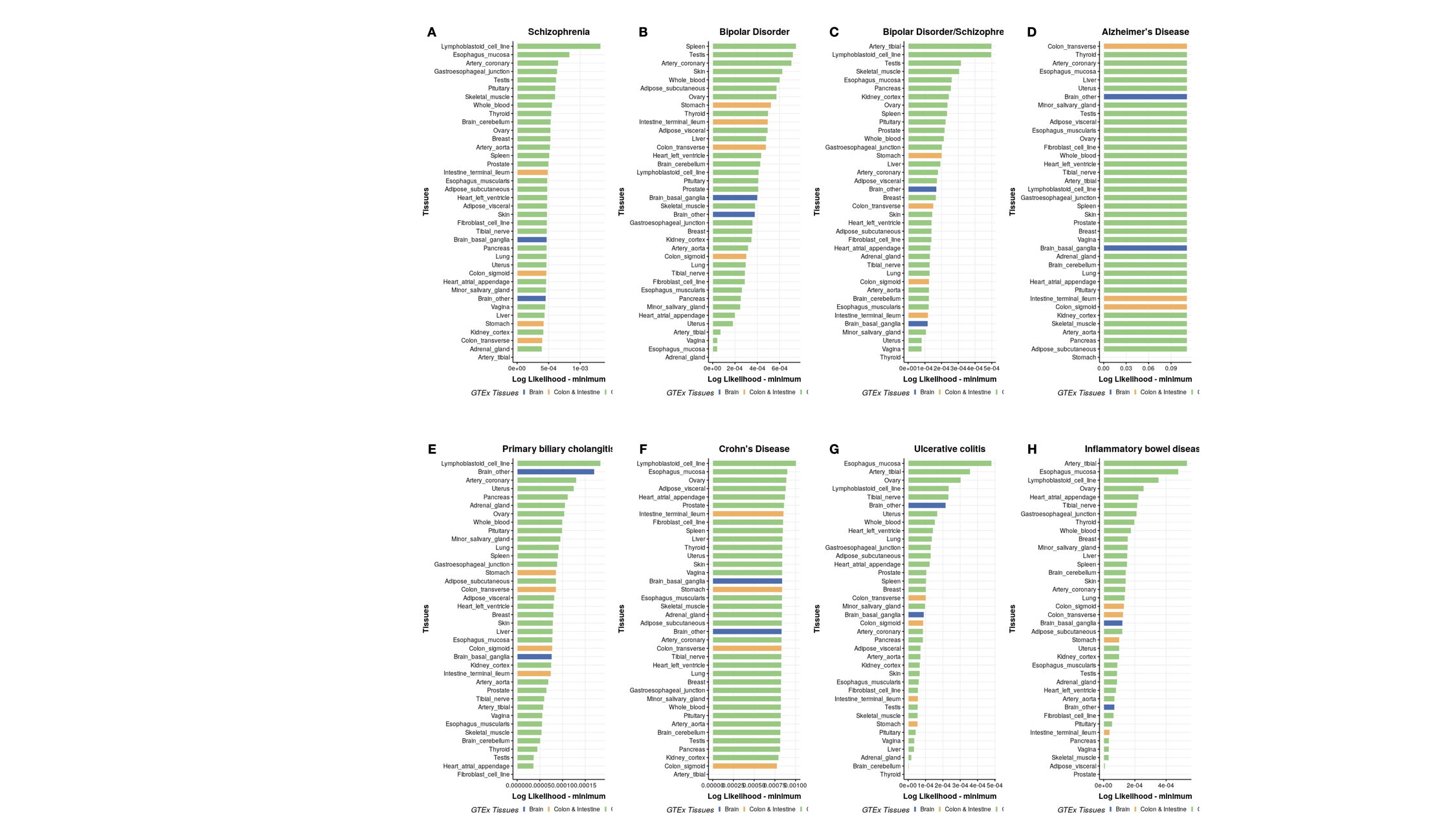

Supplement: S18 Fig — Here, based on the analysis in the main text, we filtered the edges using same procedure, but used genes that are not specific in any of the 38 tissues, and also have mean gene expression greater than 10 to build the gene co-expression network. For neurological diseases (A-D) and autoimmune diseases (E-H), we calculated the composite likelihood for each tissue, subtracted the minimum likelihood across all tissues (x-axis), and ranked traits based on these values from top to bottom in each panel (y-axis). The brain tissues are colored in blue; the colon related tissues are colored in yellow; and the rest of the tissues are colored in green. The result indicates it is important to focus on genes that are specific in at least one tissues; otherwise the results become insensible. (TIF) [file pgen.1008734.s018.tif]

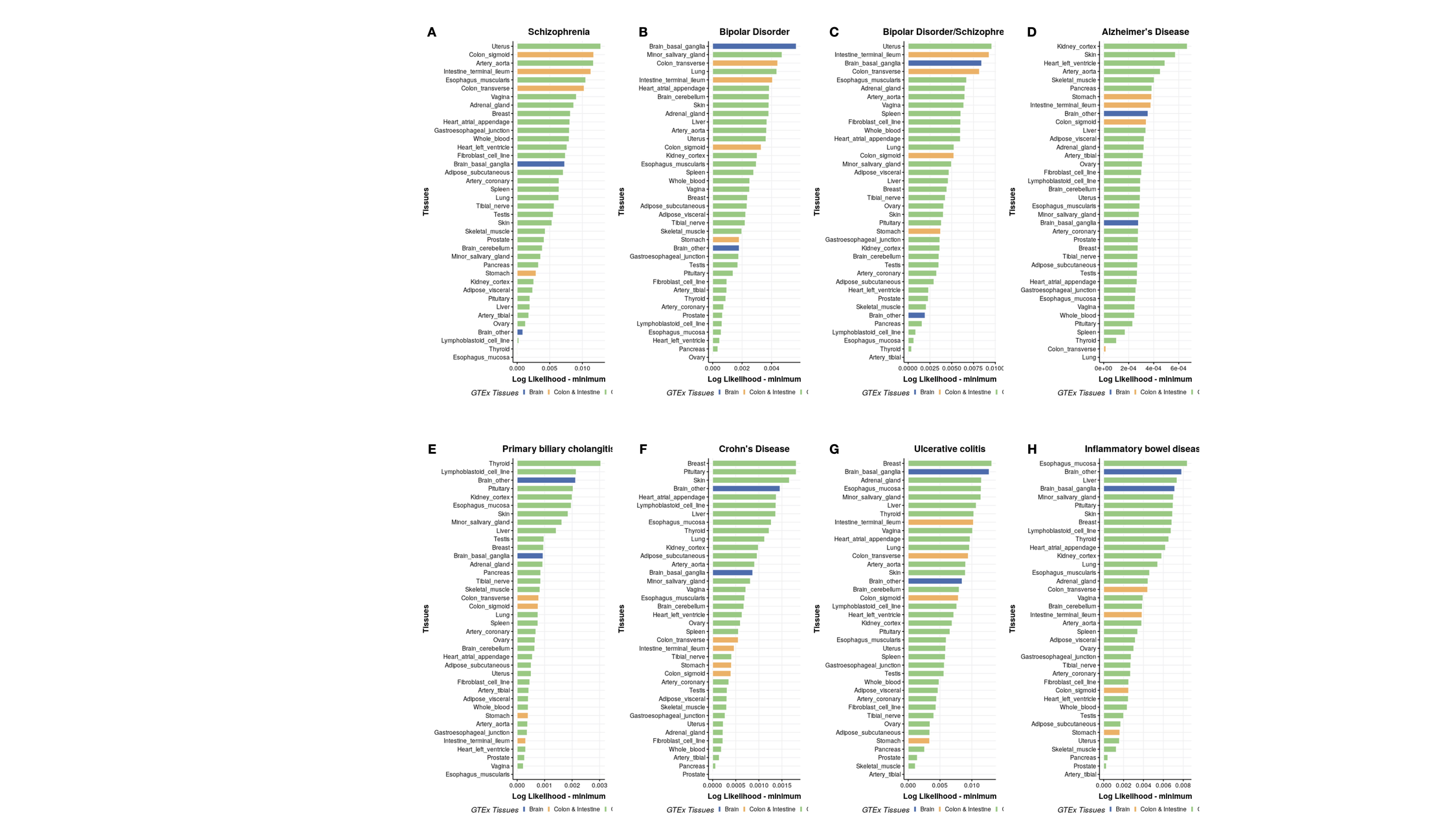

Supplement: S19 Fig — Here based on the analysis in the main text, we build the networks using the edges that are non-specific in any tissue. For neurological diseases (A-D) and autoimmune diseases (E-H), we calculated the composite likelihood for each tissue, subtracted the minimum likelihood across all tissues (x-axis), and ranked traits based on these values from top to bottom in each panel (y-axis). The brain tissues are colored in blue; the colon related tissues are colored in yellow; and the rest of the tissues are colored in green. The result indicates it is important to focus on gene pairs that are specific in at least one tissues; otherwise the results become insensible. (TIF) [file pgen.1008734.s019.tif]

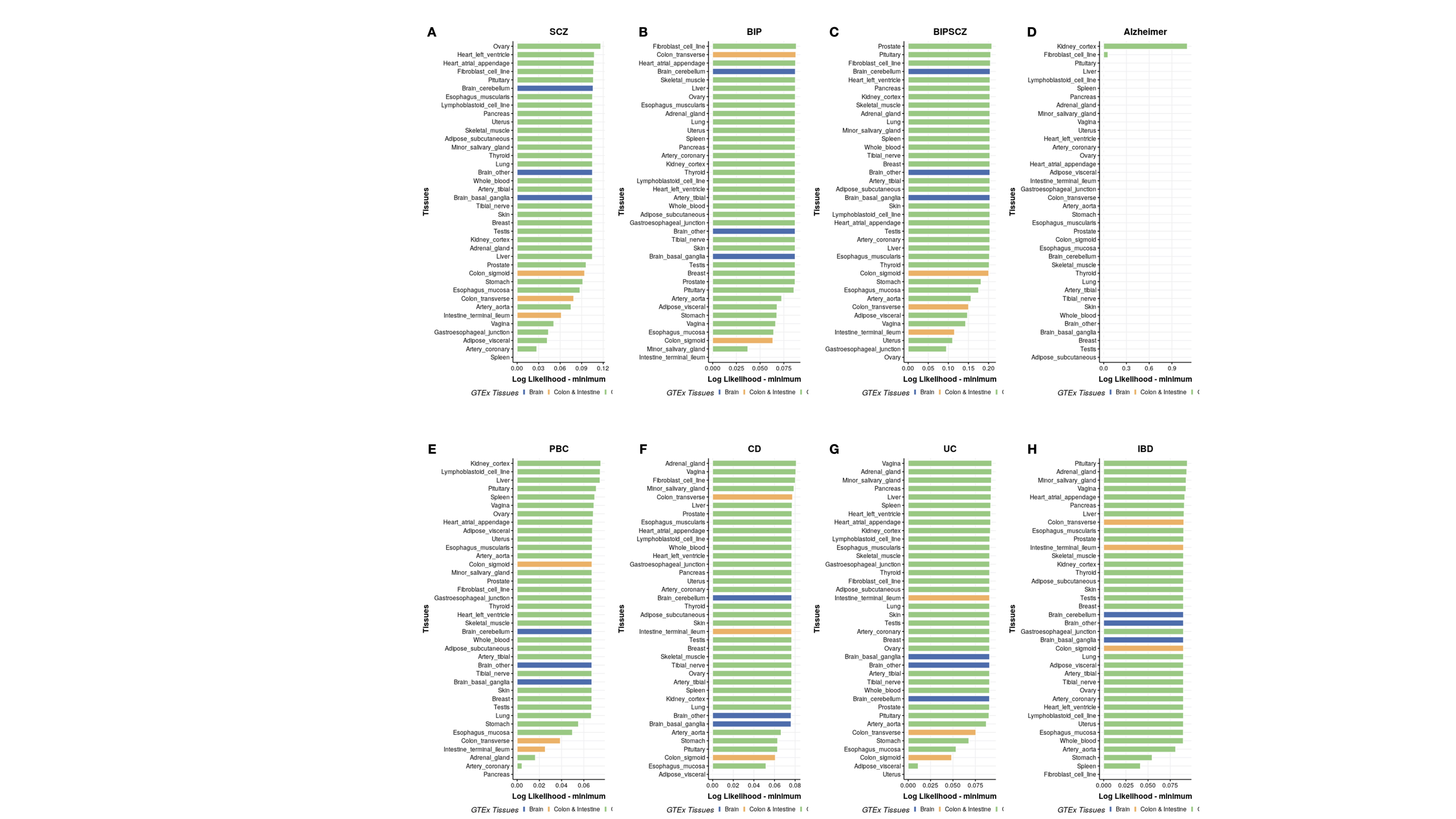

Supplement: S20 Fig — Here, based on the analysis in the manuscript, we build the tissue specific networks through WGCNA only using gene expression data. For neurological diseases (A-D) and autoimmune diseases (E-H), we calculated the composite likelihood for each tissue, subtracted the minimum likelihood across all tissues (x-axis), and ranked traits based on these values from top to bottom in each panel (y-axis). The brain tissues are colored in blue; the colon related tissues are colored in yellow; and the rest of the tissues are colored in green. The result is relatively weak compared to using networks built in PANDA. (TIF) [file pgen.1008734.s020.tif]

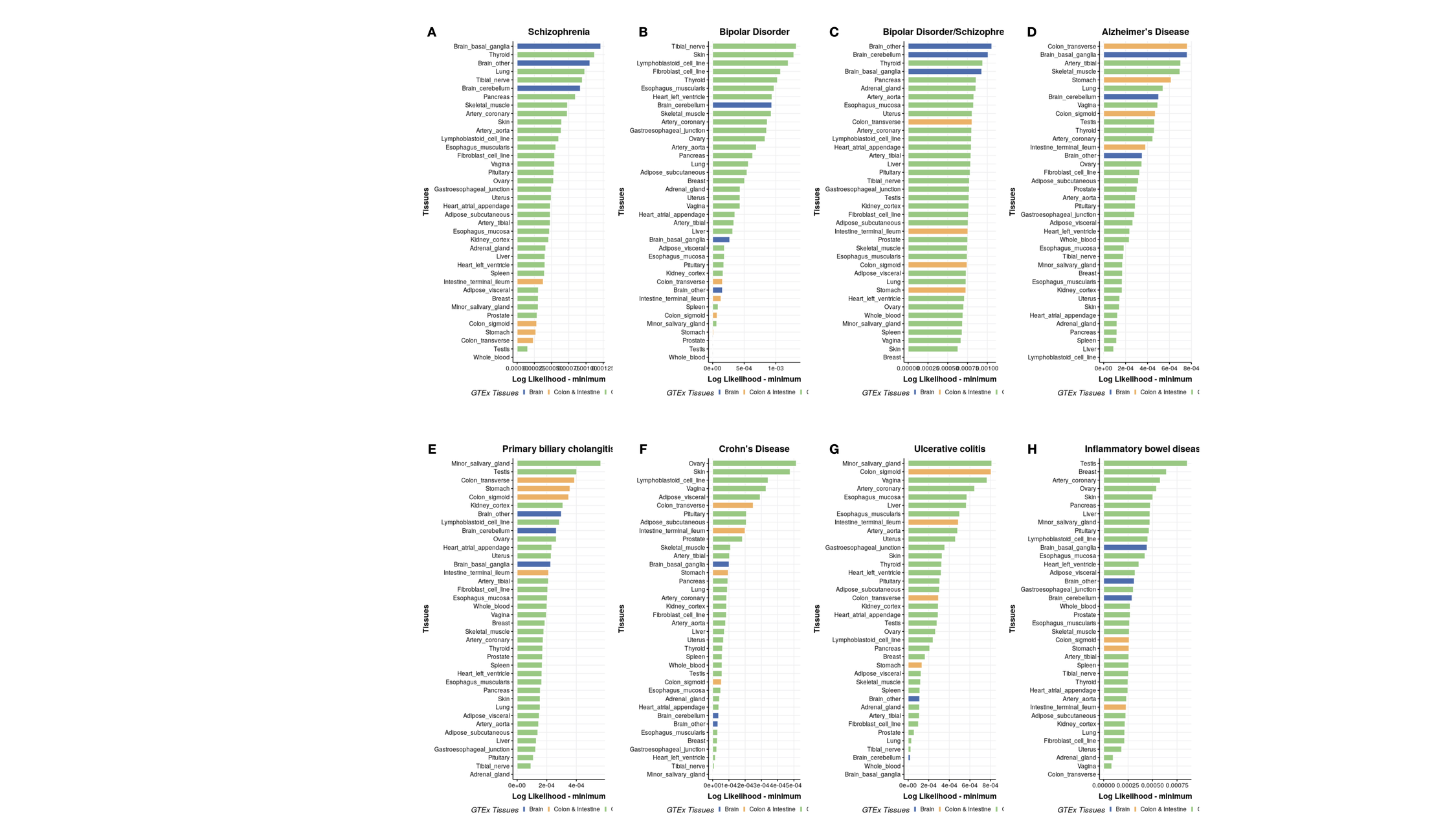

Supplement: S21 Fig — Here, based on the analysis in the main text, we used the test statistics of the per-SNP heritability as outcome in the model. For neurological diseases (A-D) and autoimmune diseases (E-H), we calculated the composite likelihood for each tissue, subtracted the minimum likelihood across all tissues (x-axis), and ranked traits based on these values from top to bottom in each panel (y-axis). The brain tissues are colored in blue; the colon related tissues are colored in yellow; and the rest of the tissues are colored in green. Consistent with the results when using per-SNP heritability as outcome, brain tissues tend to rank high for the four neurological diseases (top four panels) while colon related tissues tend to rank high for autoimmune diseases (bottom four panels). (TIF) [file pgen.1008734.s021.tif]

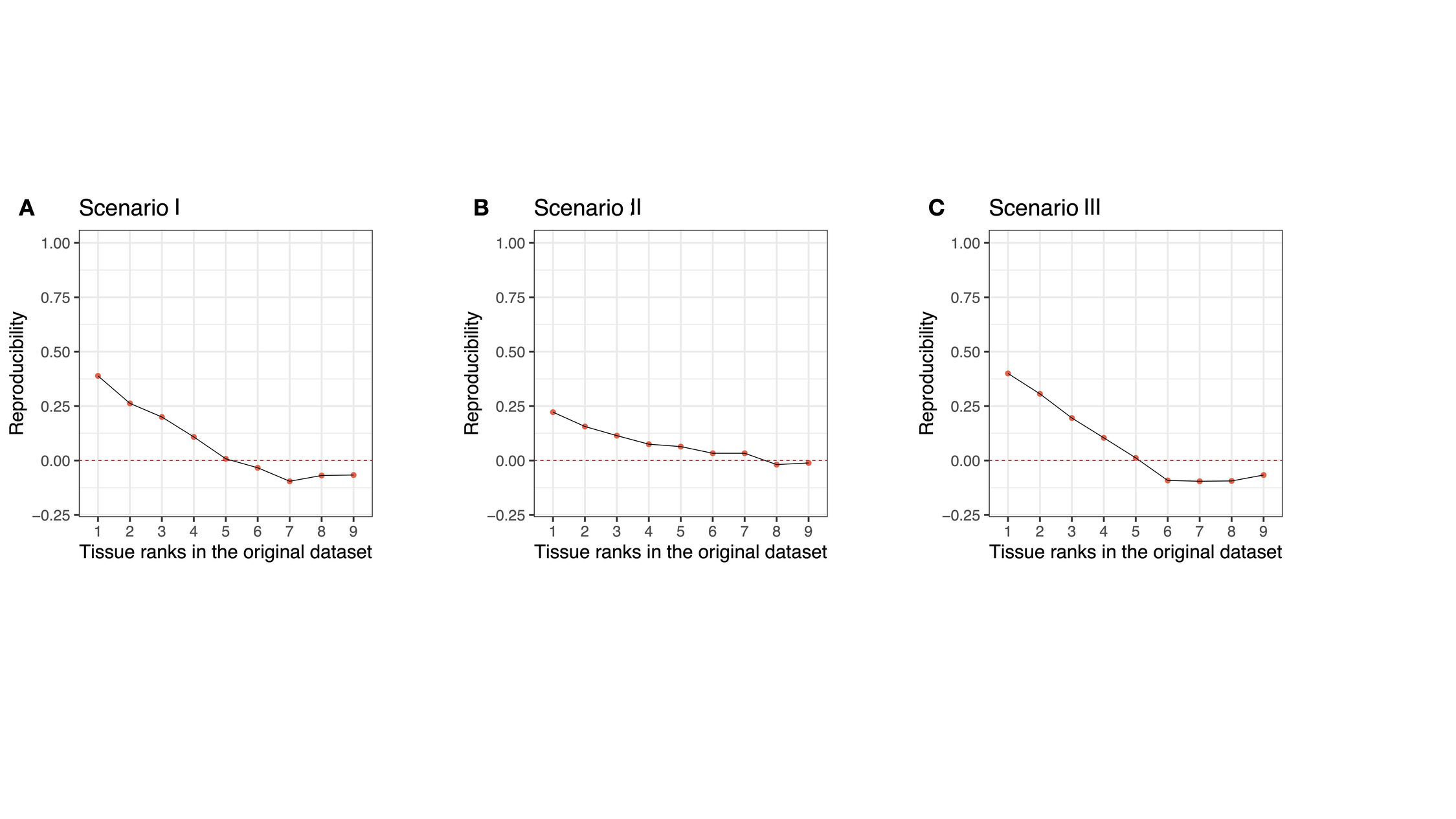

Supplement: S22 Fig — The reproducibility score (y-axis) is computed for each ordered tissue rank (x-axis) in each simulation replicate and averaged across replications. Results are shown for simulation scenarios I (A), II (B), and III (C). The reproducibility score is high for top ranked tissues while gradually reduces to zero with increasing rank order. In simulation scenario I, II, and III, we set the signal strength to be 0.02, and in scenario II and III, we set the perturbation strength to be 1%. (TIF) [file pgen.1008734.s022.tif]

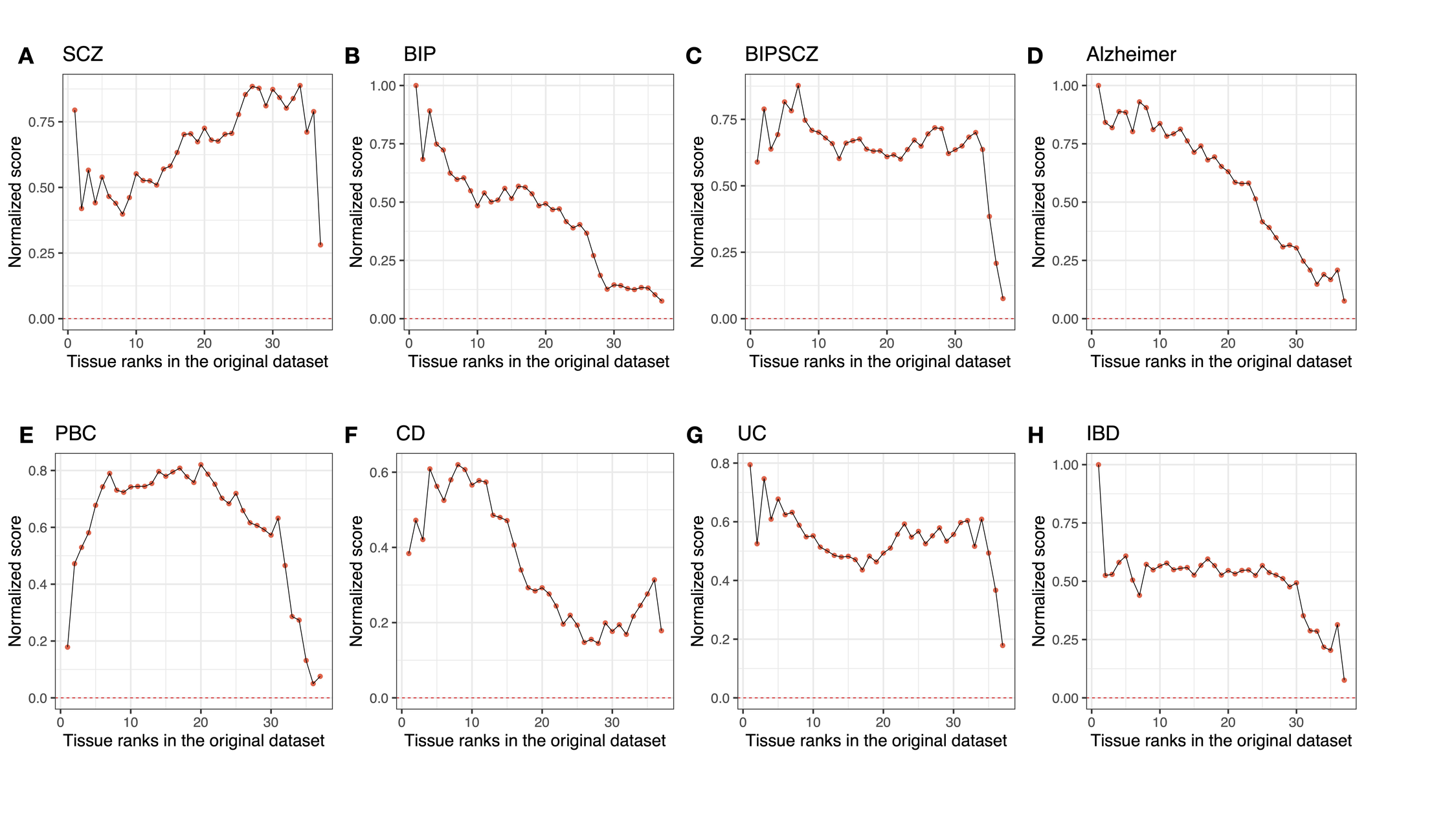

Supplement: S23 Fig — Results are shown for 4 neurological traits (A-D) and 4 autoimmune diseases (E-H). For each trait, the reproducibility score (y-axis) is computed for ordered rank (x-axis). For most traits, the reproducibility score is reasonably high for top ranked tissues. (TIF) [file pgen.1008734.s023.tif]

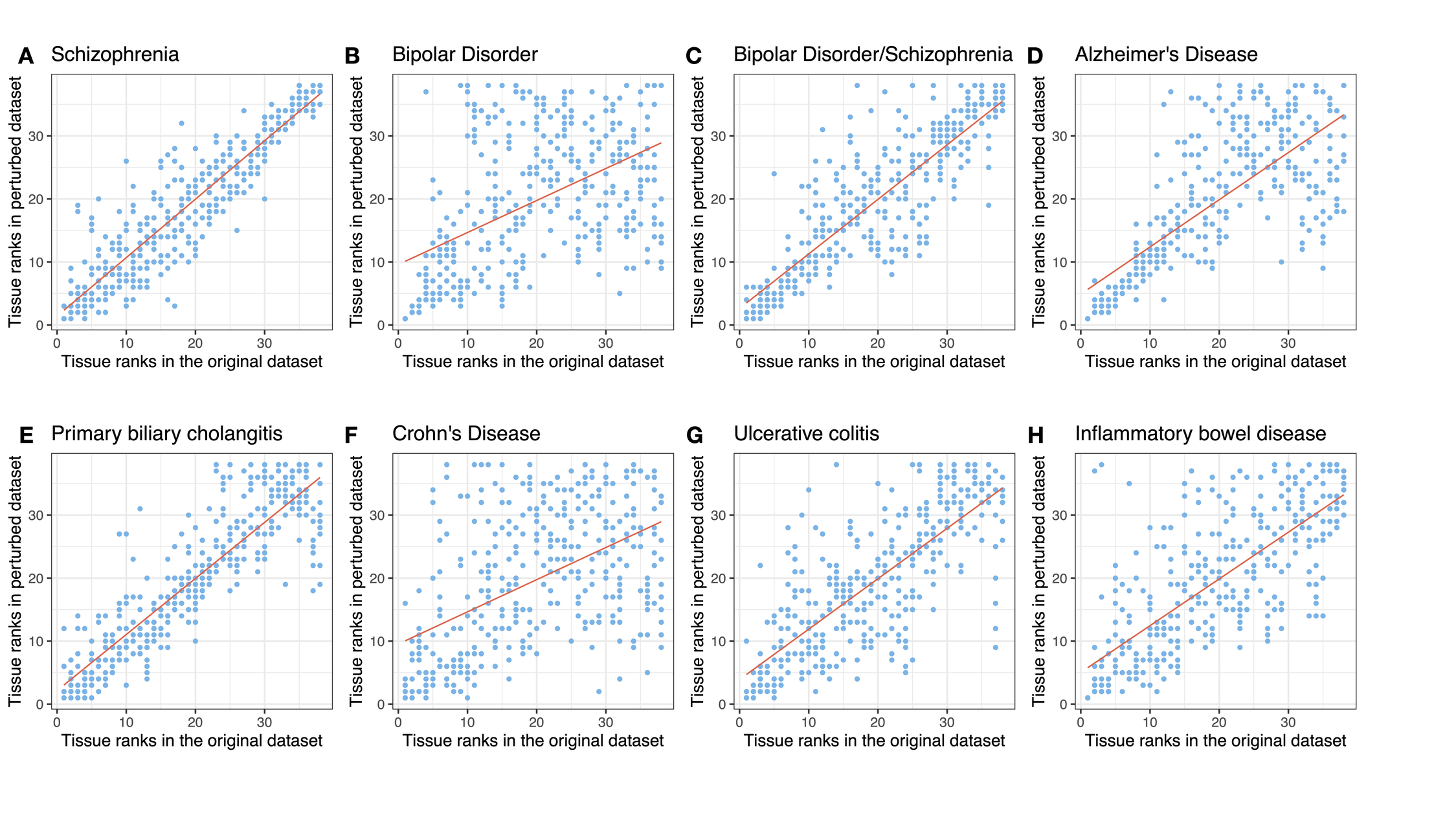

Supplement: S24 Fig — Results are shown for 4 neurological traits (A-D) and 4 autoimmune diseases (E-H). For each GWAS trait, we randomly removed 10% of connected gene pairs in the tissue specific network and constructed 10 submatrices. The rank of the tissues in the original data (x-axis) is then compared with their rank in the 10 subsampled data (y-axis). For most traits, the top ranked tissues have relatively stable rank in the subsampled data while the lowly ranked tissues have highly variable ranks in the subsampled data. (TIF) [file pgen.1008734.s024.tif]

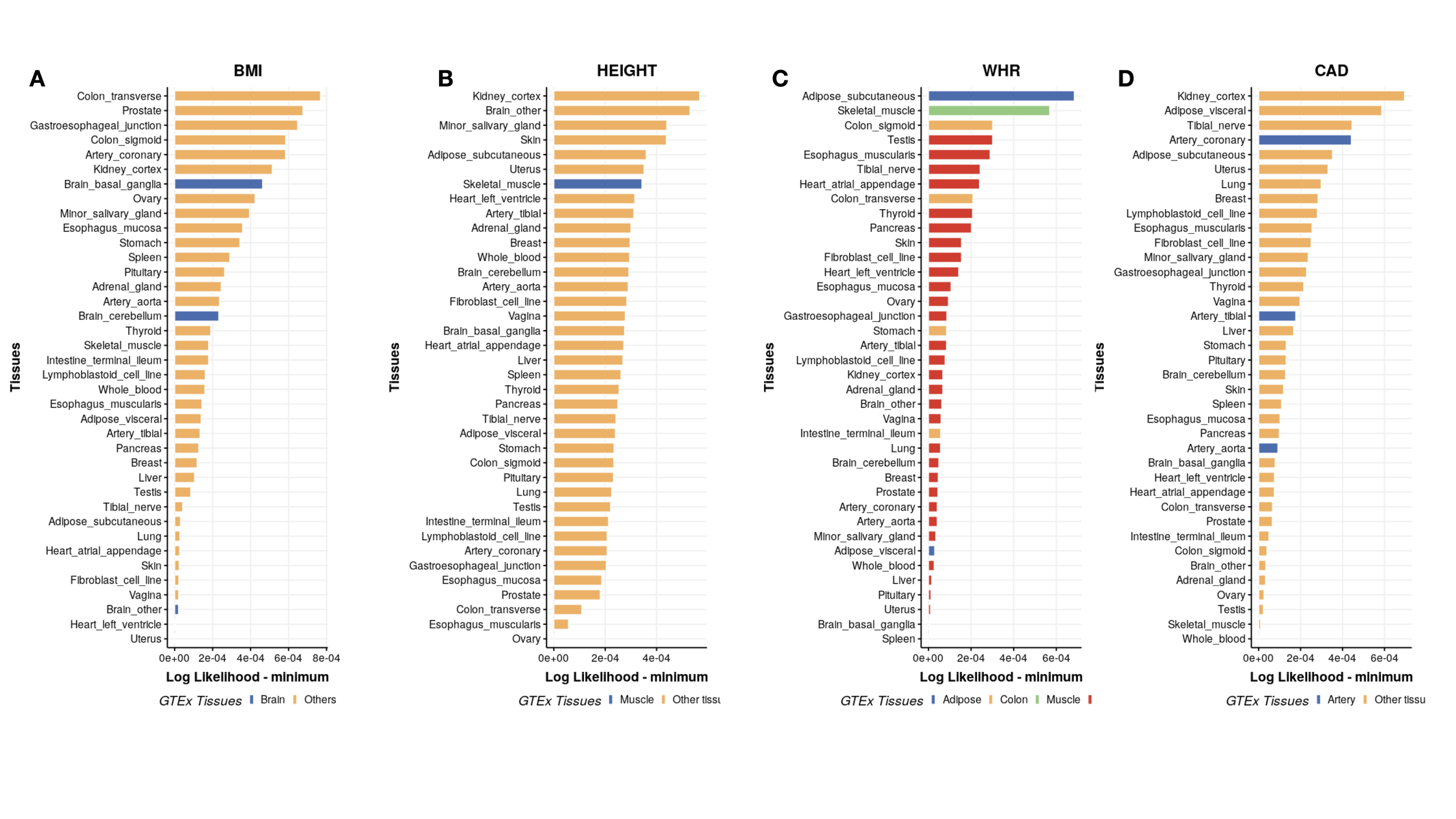

Supplement: S25 Fig — We collected additional four GWAS traits, including body mass index (BMI), height, waist-to-hip ratio (WHR) from the GIANT consortium, and Coronary Artery Disease (CAD) from the CARDIoGRAM consortium. For each GWAS trait, we calculated the composite likelihood for each tissue, subtracted the minimum likelihood across all tissues (x-axis), and ranked traits based on these values from top to bottom in each panel (y-axis). In BMI, brain tissues are colored in blue, and the other tissues are colored in yellow (A); in height, muscle tissue is colored in blue, and the other tissues are colored in yellow (B); in WHR, the adipose tissues are colored in blue, the muscle tissue is colored in green, the colon tissues are colored in yellow, and the other tissues are colored in red (C); in CAD, the artery tissues are colored in blue, and the other tissues are colored in yellow (D). The results are largely consistent with previous literatures. (TIF) [file pgen.1008734.s025.tif]

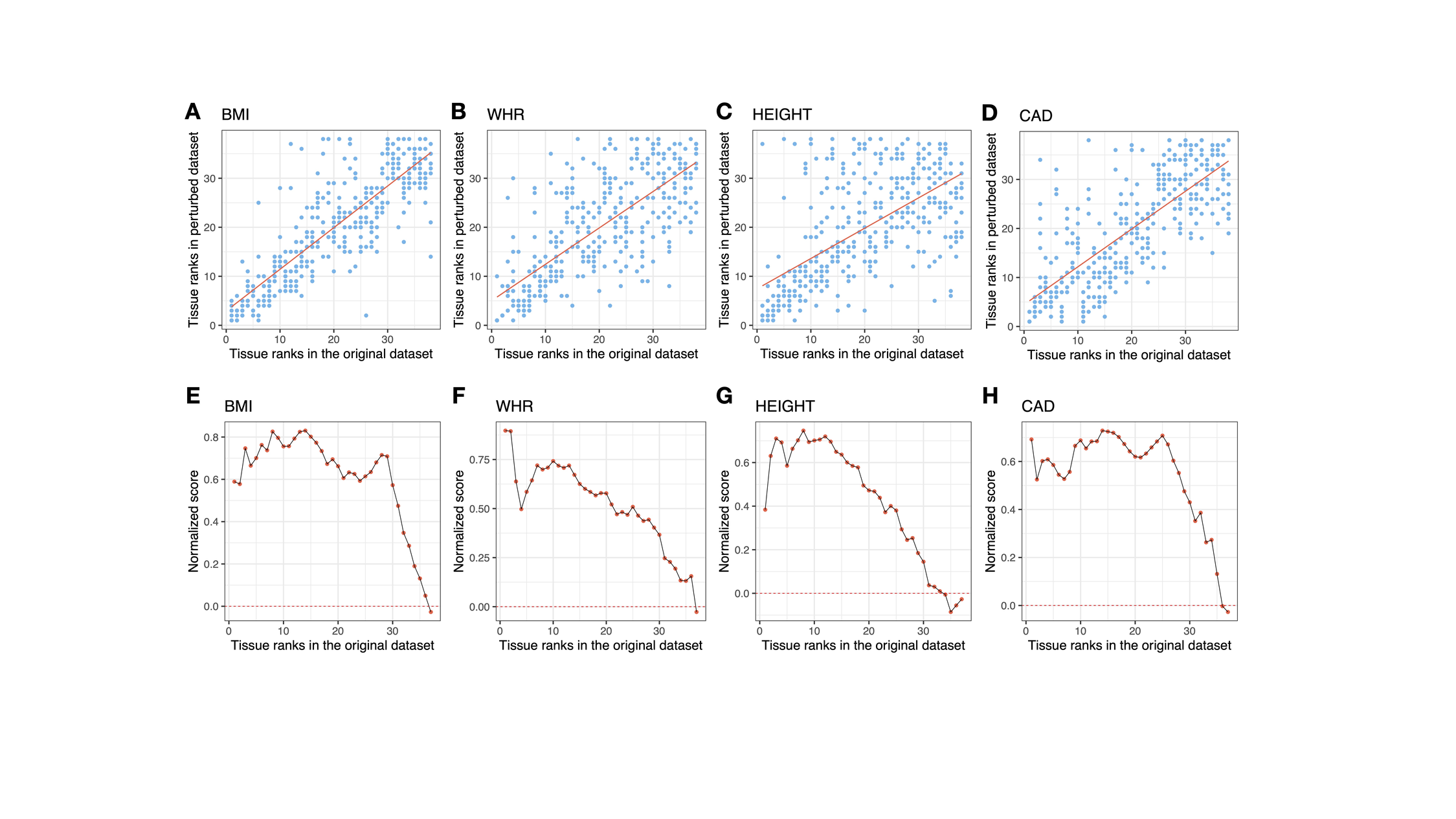

Supplement: S26 Fig — Results are shown For each of the additional four GWAS trait including body mass index (BMI), height, waist-to-hip ratio (WHR) from the GIANT consortium, and Coronary Artery Disease (CAD) from the CARDIoGRAM consortium in (A-D). For each GWAS trait, we randomly removed 10% of connected gene pairs in the tissue specific network and constructed 10 submatrices. The rank of the tissues in the original data (x-axis) is then compared with their rank in the 10 subsampled data (y-axis). For most traits, the top ranked tissues have relatively stable rank in the subsampled data while the lowly ranked tissues have highly variable ranks in the subsampled data. The Reproducibility score for 38 tissues in the real data are shown in (E-H). For each trait, the reproducibility score (y-axis) is computed for ordered rank (x-axis). For most traits, the reproducibility score is reasonably high for top ranked tissues. (TIF) [file pgen.1008734.s026.tif]
