# Supplementary material for: Leveraging gene co-expression patterns to infer trait-relevant tissues in genome-wide association studies
Source: PLoS Genet. 2020 Apr 20;16(4):e1008734. doi: 10.1371/journal.pgen.1008734 (PMC7192514; doi:10.1371/journal.pgen.1008734)
Supplement: S1 Text — (DOCX) [file pgen.1008734.s033.docx]

**Supplementary Text**

**CoCoNet Inference**

Our composite likelihood for the general version of covariance regression network model is defined based on the product of pair-wise likelihood. For each pair of genes $i$ and gene $j$, we have:

$$\left( \begin{matrix} y_{i} \\ y_{j} \end{matrix} \right)\sim BN(\left( \begin{matrix} \mu\\ \mu\end{matrix} \right),\sum_{k=0}^{K} \sigma_{k}^{2}\left( \begin{matrix} a_{ii}^{\left( k \right)} & a_{ij}^{\left( k \right)} \\ a_{ij}^{\left( k \right)} & a_{jj}^{\left( k \right)} \end{matrix} \right))$$

where ***A*** is the adjacency matrix, and $\boldsymbol{A}^{k}=(a_{ij}^{\left( k \right)})$ is the *k-*th power of $\boldsymbol{A}$, for any integer *k*.

We can write $\sum_{k=0}^{K} \sigma_{k}^{2}\boldsymbol{A}^{k}=\left( \begin{matrix} b_{ii} & b_{ij} \\ b_{ji} & b_{jj} \end{matrix} \right)$,

where

$$b_{ii}=b_{jj}=\sigma_{0}^{2}+\sigma_{1}^{2}a_{ii}^{(1)}+ \sigma_{2}^{2}a_{ii}^{(2)}+\ldots\sigma_{K}^{2}a_{ii}^{\left( K \right)}=\sigma_{0}^{2}$$

since we set $a_{ii}^{\left( k \right)}$ to be zero for $k\geq1$,

and

$b_{ij}=b_{ji}=\sigma_{1}^{2}a_{ij}^{(1)}+ \sigma_{2}^{2}a_{ij}^{(2)}+\ldots\sigma_{K}^{2}a_{ij}^{(K)}$.

The likelihood function for a pair of gene $i$ and $j$:

$$\log P\left( (y_{i},y_{j}|\mu,\sigma_{0}^{2},\cdots,\sigma_{k}^{2}) \right)=-\frac{1}{2}\log\left( b_{ii}^{2}-b_{ij}^{2} \right)-\frac{1}{2\left( b_{ii}^{2}-b_{ij}^{2} \right)}\left( \begin{matrix} y_{i}-\mu\\ y_{j}-\mu\end{matrix} \right)^{T}\left( \begin{matrix} b_{jj} & -b_{ij} \\ -b_{ji} & b_{ii} \end{matrix} \right)\left( \begin{matrix} y_{i}-\mu\\ y_{j}-\mu\end{matrix} \right)-\frac{1}{2}\log\left( \frac{1}{2\pi} \right)=-\frac{1}{2}\log\left( b_{ii}^{2}-b_{ij}^{2} \right)-\frac{1}{2\left( b_{ii}^{2}-b_{ij}^{2} \right)}[\left( \left( y_{i}-\mu\right)^{2}+\left( y_{j}-\mu\right)^{2} \right)b_{ii}-2b_{ij}\left( y_{i}-\mu\right)\left( y_{j}-\mu\right)]-\frac{1}{2}\log\left( \frac{1}{2\pi} \right)$$

Full composite likelihood function:

$$l\left( \theta\right)=\sum_{i=1}^{m}\Sigma_{j>i}^{m}logP(y_{i},y_{j}|\mu,\sigma_{0}^{2},\cdots,\sigma_{k}^{2})$$

$$\log P=\frac{1}{2\left( m-1 \right)}\sum_{i=1}^{m} \sum_{j\neq i} logP(y_{i},y_{j}|\mu,\sigma_{0}^{2},\cdots,\sigma_{k}^{2})=\frac{1}{2(m-1)}\sum_{i=1}^{n} \sum_{j\neq i} [-\frac{1}{2}\log\left( b_{ii}^{2}-b_{ij}^{2} \right)-\frac{1}{2\left( b_{ii}^{2}-b_{ij}^{2} \right)}[\left( \left( y_{i}-\mu\right)^{2}+\left( y_{j}-\mu\right)^{2} \right)b_{ii}-2b_{ij}\left( y_{i}-\mu\right)\left( y_{j}-\mu\right)]-\frac{1}{2}\log\left( \frac{1}{2\pi} \right)]$$

**Reproducibility Score**

Here, we aim to quantify the uncertainty of tissue rank in the CoCoNet output. We do so by using a sub-sampling/perturbation based strategy. Specifically, we performed subsampling on each tissue specific adjacency matrix by randomly removing ten percent of the connected gene pairs. We then applied CoCoNet to the subsampled matrices and rank tissues based on the subsampled data. We repeated the whole process ten times. We asked how consistent the rank of a given tissue is between the original data and the subsampled data. In particular, we asked what’s the proportion of times the rank one tissue in the original data is also ranked as the first in these subsampled data; and more generally, what’s the proportion of times the rank *k* tissue in the original data also has a rank of *k* or higher in the subsampled data. Technically, assume we have *K* tissues in total, we denote ${S_{k}}_{1}$ as the set of top *k* tissues in the original data and denote ${S_{k}}_{2}$ as the set of tissues that is among the top *k* tissues from at least one of the ten subsampled data. We then computed a score of the ranked list by computing the average value of $s=\left| {S_{k}}_{1} \cap S_{k2} \right|/k$ following [1]. The score s for the top k tissue computed this way is constrained to between 0 and 1 and has an expectation of $k/K$ if tissue ranking is completely randomly. Therefore, we normalized the score by computing $(s-k/K)/(1-k/K)$ (in line with the definition of the commonly used kappa statistics). The normalized score is constrained to be below 1 and is positive only when the ranking is reproducible beyond what is expected by chance alone. We denote the normalized score as the reproducibility score, which provides an uncertainty quantification of tissue ranking. Intuitively, if the inference results are sensitive to the misspecification of adjacency matrices, then we would expect to see a low reproducibility score close to be zero (or negative). In contrast, if the inference results are not sensitive to the misspecification of adjacency matrices, then we would expect to see a high reproducibility score close to 1.

**Controlling for Distance Confounding**

Distance between the pairs of genes may confound our analysis results. We controlled for such confounding by adding the gene pair-wise distance matrix as an additional term in CoCoNet:

For each pair of genes $i$ and $j$, we consider the pair-wise likelihood $P(y_{i},y_{j}|\mu,\sigma_{k}^{2})$ as

| $\left( \begin{matrix} y_{i} \\ y_{j} \end{matrix} \right)\sim BN(\left( \begin{matrix} \mu\\ \mu\end{matrix} \right),\sigma_{0}^{2}\boldsymbol{I}+\sigma_{1}^{2}\left( \begin{matrix} a_{ii} & a_{ij} \\ a_{ij} & a_{jj} \end{matrix} \right)+\sigma_{D}^{2}\left( \begin{matrix} d_{ii} & d_{ij} \\ d_{ij} & d_{jj} \end{matrix} \right))$ |  |
| --- | --- |

where $\mu$ is the intercept; *BN* denotes a bivariate normal distribution; the constructed tissue-specific symmetric adjacency matrix $\boldsymbol{A}=(a_{ij})$, where its *ij*’th element $a_{ij}$ is one $if gene i is connected to gene j in the network$ and is zero otherwise; $a_{ii}$ is set to be zero for any $1\leq i\leq m$ to ensure the absence of self-loops [2]; $\boldsymbol{D}=(d_{ij})$ is the gene distance matrix, where its *ij*’th element $d_{ij}$ is the distance measured between gene $i$ and gene $j$ based on their in transcript starting site (TSS) divided by the maximum gene pair distance; we also set $d_{ij}=1$ if the two genes are on different chromosomes. Intuitively, $\sigma_{1}^{2}\boldsymbol{A}$ captures the gene-level effect size correlation due to gene co-expression connections among genes, while $\sigma_{D}^{2}\boldsymbol{D}$ captures the gene-level effect size similarity due to distance. Therefore, by controlling for the additional term $\sigma_{D}^{2}\boldsymbol{D}$, we can control for distance confounding.

**Ising Model**

Let $X=\{X_{1},\ldots, X_{m}\}$ be a set of random variables $X_{j}\in\{0,1\}$ in a discrete set to denote the outcome. Let $G=(X,E)$ be an undirected graph with edge set $E \subset X\times X$. In the Ising model, the pseudolikelihood function is given by the product of local conditional densities, and it takes the form:

$$L_{p}\left( \theta\right)=\prod_{i=1}^{m} L_{p}\left( x_{j}|x_{-j} \right)=\prod_{j=1}^{m} \frac{\exp\left( H_{j}x_{j}+x_{j}{\sum_{k\neq j} J}_{jk}x_{k} \right)}{1+\exp\left( H_{j}x_{j}+x_{j}{\sum_{k\neq j} J}_{jk}x_{k} \right)}$$

where the $H$’s and $J$’s are marginal and interaction parameters of the model. To avoid the computational inefficiency for inferring maximum likelihood estimator on a graphical model, we used the maximum pseudolikelihood estimator (MPLE). We use the Nelder-Mead method implemented in the optim function in R to maximizes the above pseudolikelihood function.

1. Boulesteix AL, Slawski M. Stability and aggregation of ranked gene lists. Brief Bioinform. 2009;10(5):556-68. Epub 2009/08/15. doi: 10.1093/bib/bbp034. PubMed PMID: 19679825.

2. Urry MJ, Sollich P. Random Walk Kernels and Learning Curves for Gaussian Process Regression on Random Graphs. J Mach Learn Res. 2013;14:1801-35. PubMed PMID: WOS:000323367000005.
